# Identifying Developmental Zones in Maize Lateral Root Cell Length Profiles using Multiple Change-Point Models

**DOI:** 10.3389/fpls.2017.01750

**Published:** 2017-10-26

**Authors:** Beatriz Moreno-Ortega, Guillaume Fort, Bertrand Muller, Yann Guédon

**Affiliations:** ^1^LEPSE, INRA, Montpellier SupAgro, Montpellier, France; ^2^CIRAD, UMR AGAP, Montpellier, France; ^3^Inria, Virtual Plants, Montpellier, France; ^4^Université de Montpellier, Montpellier, France

**Keywords:** auxin mutant, lateral root diversity, multiple change-point model, piecewise linear function, principal component analysis, root apex

## Abstract

The identification of the limits between the cell division, elongation and mature zones in the root apex is still a matter of controversy when methods based on cellular features, molecular markers or kinematics are compared while methods based on cell length profiles have been comparatively underexplored. Segmentation models were developed to identify developmental zones within a root apex on the basis of epidermal cell length profiles. Heteroscedastic piecewise linear models were estimated for maize lateral roots of various lengths of both wild type and two mutants affected in auxin signaling (*rtcs* and *rum-1*). The outputs of these individual root analyses combined with morphological features (first root hair position and root diameter) were then globally analyzed using principal component analysis. Three zones corresponding to the division zone, the elongation zone and the mature zone were identified in most lateral roots while division zone and sometimes elongation zone were missing in arrested roots. Our results are consistent with an auxin-dependent coordination between cell flux, cell elongation and cell differentiation. The proposed segmentation models could extend our knowledge of developmental regulations in longitudinally organized plant organs such as roots, monocot leaves or internodes.

## Introduction

Since the pioneering studies of Sachs (1873) and Darwin (1880), the root apex has been one of the most widely used plant organs to study cell division, cell elongation and cell differentiation which occur within successive zones (Goodwin and Stepka, 1945; Erickson and Sax, 1956b). While the longitudinal cellular pattern within the root apex and the naming of the different zones are now the matter of tentative consensus views (Barrio et al., 2013; Ivanov and Dubrovsky, 2013), there is still no general agreement regarding the criteria used to define the limits between these zones (Verbelen et al., 2006; Ivanov and Dubrovsky, 2013; Bizet et al., 2015). Historically, the shootward limit of the division zone (also called the root apical meristem, according to Barrio et al., 2013; Ivanov and Dubrovsky, 2013, where cells divide at various rates and in different proportions) was identified by the presence/absence of mitotic figures in longitudinal sections (Clowes, 1959; Hejnowicz, 1959). By the turn of the last century, molecular markers have revolutionized the histology and, regarding cell division, cyclins which show marked overexpression at precise time points during the cell cycle have been used (Ferreira et al., 1994; West et al., 2004). Such type of discrete labeling leads to a probabilistic pattern and this has allowed the identification of a transition zone in the shootward region of the root apical meristem. In this zone, a progressive decrease of the occurrence of cell division is observed while cells acquire the capacity to elongate through vacuolization (Baluška et al., 1992) and cortical microtubule reorganization (Baluška et al., 1996; Baskin et al., 1999). After the transition zone, cells move to a rapid elongation zone and, to our knowledge, there is no consensus molecular marker for this zone although some members of the expansion gene family show tight association of their expression with elongation rate in monocot leaves (Muller et al., 2007) or internodes (Lee and Kende, 2001). Growth cessation at the shootward limit of the elongation zone has been associated with cell wall stiffening (Tomos and Pritchard, 1994), peroxidase activity (though more convincingly in aerial organs, see MacAdam et al., 1992) and the burst of reactive oxygen species (Dunand et al., 2007) but none of these events were used as marker to locate this limit. Moreover, the formation of a root hair bulge has often been taken as a marker of the switch from elongation to differentiation although this tends to occur before the end of the elongation zone (Ma et al., 2003; Le et al., 2004).

Alternative to cellular features or molecular markers are kinematic studies (Sharp et al., 1988; Muller et al., 1998; Walter et al., 2002). They are based on the combination of non-destructive observations of the spatial distribution of local growth within the root apex with the cell length profile in the same zone (Erickson and Sax, 1956b). These techniques quantify the cell division rate and locate the shootward limit of the division zone by using an analogous of the continuity equation used in fluid mechanics here applied to local cell density (Erickson and Sax, 1956b; Beemster and Baskin, 1998; Muller et al., 1998). However, averaging local growth profiles for several roots was identified as a source of bias, leading to smooth rapid individual variations and probably to overestimate the size of the division zone (van der Weele et al., 2003). Moreover, when growth is non-stationary, this technique requires the incorporation of a time-dependency term (Silk, 1992; Beemster and Baskin, 1998) which further adds uncertainties.

Developmental zones can also be identified in root apices on the basis of cell length profiles alone. Meristematic cells are short in length. The exit from the cell cycle and the entry into the elongation zone are characterized by a rapid increase in cell length while the end of the elongation zone is expected to correspond to cell length reaching a plateau. Different methods have been used to determine meristem length based on cell length profiles including expert visual methods (Casamitjana-Martinez et al., 2003; Mouchel et al., 2004), empirical thresholds on cell length (Beemster et al., 2002) and geometrical approaches to detect change points (French et al., 2012). These methods are well suited to simple root structures such as in *Arabidopsis thaliana* where only a few cells per files are present and abrupt changes in length can be easily detected. It becomes more difficult when larger, more complex roots, with many cell files are considered or when not all the cells can be visible from the quiescent center, hampering the use of methods based on cell indices (French et al., 2012). Recently, a method was proposed that delineates complex root meristems based on a statistically-defined cell length threshold (Bizet et al., 2015). This method proved to be robust although it does not allow to detect patterns within the meristem (such as gradual increase or decrease in cell length or change points) whereas these can be useful information when analyzing the impact of environmental stresses or genetic effects. Moreover, none of these methods were designed to identify the limit between the elongation zone and the mature zone. Very few attempts have been made in this direction because of the difficulty to deal with the large standard deviation of cell length around this limit (Silk et al., 1989).

A generic segmentation method, (i) based on cell length profiles, (ii) relying on minimum *a priori* biological assumptions, (iii) applicable to a large diversity of root growth dynamics, and (iv) able to detect limits both between the division zone and the elongation zone and (v) between the elongation zone and the mature zone is thus missing. The aim of this study was to propose such a generic method for identifying root developmental zones in cell length profiles. We used heteroscedastic piecewise Gaussian linear models (i.e., with a residual variance specific to each developmental zone; Hawkins, 1976). These specific multiple change-point models are distinct from segmented regression or broken-line models (Muggeo, 2003) which are constrained to be homoscedastic (i.e., with a residual variance common to the different developmental zones). This assumption appeared to be unrealistic given the large changes in variance along root cell length profiles (Goodwin and Stepka, 1945; Pritchard et al., 1990). As a biological material, we choose maize lateral roots showing a large diversity of lengths and diameters, likely corresponding to various growth dynamics with acceleration, deceleration and rapid growth arrest as reported in other species (Freixes et al., 2002). These dynamics were expected to correspond to meristem enlargement, shrinkage or exhaustion, respectively (Dubrovsky and Gómez-Lomeli, 2003; Sánchez-Calderón et al., 2005). In order to increase the sources of variability in our lateral root sample, and given the known impact of auxin on the establishment and the maintenance of the root meristem (Pacifici et al., 2015) and on the balance between cell division and differentiation (Dello Ioio et al., 2008), we used two independent maize mutants altered in auxin signaling: *rtcs* codes for a LOB-domain transcription factor and carries auxin-responsive elements in its promoter (Taramino et al., 2007) while *rum1* codes for an AUX/IAA protein (Woll et al., 2005). Both mutants are also defective in seminal root formation. The objectives of this work were thus twofold: (i) design statistical models for identifying development zones in cell length profiles observed in root apices and (ii) on this basis, identify emerging properties in particular in terms of coupling/uncoupling between cell division, elongation and differentiation processes and characterize the intrinsic modulation of the root developmental pattern for a large diversity of lateral roots as well as the impact of a perturbation in auxin signaling.

## Materials and methods

### Plant material, growth conditions, and lateral root apex sampling

Maize (*Zea mays* L.) seeds of the hybrid B73xUH007 (referred to as wild type in the following) used in this study were produced within the European FP7 project EURoot (http://www.euroot.eu). Seeds of *rtcs* (Taramino et al., 2007) and *rum-1* (Woll et al., 2005) maize mutants, both in the B73 background, were provided by Frank Hochholdinger (University of Bonn, Germany). Germinated seeds were transferred upon emergence of the radicle on the top of 70 × 40 cm rhizotrons adapted from Neufeld et al. (1989). Root systems were allowed to develop between a layer of cellulose acetate tissue in contact with nutrient and water rich compost and a slide of plexiglass. Rhizotrons were installed into 1 m^2^ growth chambers under controlled conditions (24/20°C day/night temperature, 14 h photoperiod, 1 kPa VPD, and PPFD of 300 μmol photons/m^2^/s).

**Figure 1:**
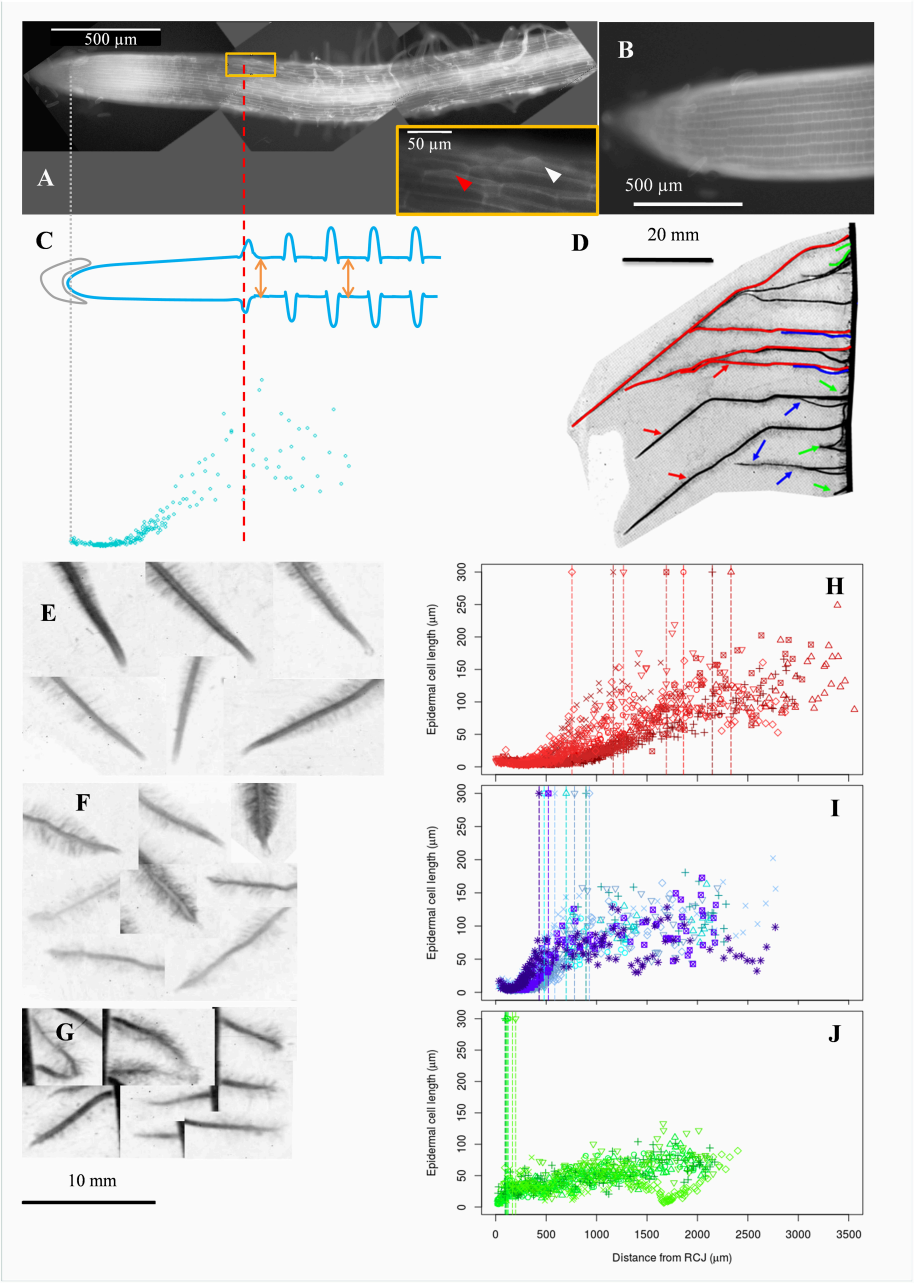
Acquisition of epidermal cell length profiles, diversity of maize lateral roots and examples of cell length profiles collected in roots of different types. **(A)** Autofluorescence microphotography of a maize lateral root apex obtained as a composite of 3 different microscopy images (black background). Arrowheads in the inbox indicate root hair bulges. The most rootward epidermal cell with a visible root hair bulge is indicated by a red arrowhead. **(B)** Zoom on a meristematic zone. **(C)** Epidermal cell lengths (blue points) were sampled along the longitudinal axis of the root. The positions of the root cap junction (gray line; origin of the longitudinal axis) and of the most rootward root hair bulge (red line) were recorded. Root diameter was sampled at two different positions beyond the first root hair (orange arrows). **(D)** Typical display of lateral roots along a maize primary root. Lateral roots were visually classified in 3 categories depending on length at a given insertion point and distance from tip to first root hair, indicative of root growth rate. Red, green, and blue refer respectively to long and vigorous roots (type A), short (<10 mm) and arrested roots (type C) and intermediate, slow growing roots (type B). **(E–G)** Examples of lateral root tips sampled from the three types. Types B and C had visually short distances between tip and first root hair position. **(H–J)** Examples of cell length profiles from lateral roots belonging to the three types shown in **(E–G)** respectively. Data from 4 to 7 individual roots are merged. Vertical dotted lines mark the position of the first root hair bulge visible in fluorescence images.

After 2 weeks, a selection of approximately 1 cm long root apices from 42 lateral roots encompassing the diversity of roots present along the primary root of several replicate plants was harvested. The lateral roots were sampled within 3 categories based on (i) the root length relative to its neighbors (Figure 1D), and (ii) the proximity of the first root hair to the root tip (Figures 1E–G) which is known to be a good indicator of the elongation rate (Watt et al., 2003; Pagès et al., 2010), being more or less closely associated with the end of the elongation zone (Le et al., 2001). We named these roots A, B and C with type A roots being long, type C roots being very short (<1 cm) and type B roots being intermediate in length. This typology matches that recently identified in pearl millet on the basis of anatomical features (Passot et al., 2016). Using independent experiments (not described here), we evaluated the growth patterns of these roots and found that type A root growth rate was high (8–17 mm day^−1^) and increases markedly the first days of growth by contrast with type B and C roots which show slow and fast deceleration, ending up in growth arrest in (on average) 6 and 3 days respectively. In the two mutants, roots of similar types were found although long roots were globally shorter than in the wild type. In addition, vigorous lateral roots emerging from curvatures of the primary roots induced by the alteration of gravitropism were categorized as A′. Each of these root types were present in all individual plants and our sampling protocol aimed at capturing the largest diversity, including that due to plant vigor.

### Image analysis and acquisition of lateral root cell length profiles and morphological properties

Root apices were placed in a fixative solution of 1:3 vol/vol acetic acid: 70% ethanol and stored at 4°C. After 2 days, the fixed material was moved to a clearing solution of chloral hydrate (200 g chloral hydrate in 20 ml glycerol and 30 ml water) for at least 4 h (Wuyts et al., 2010). Roots were mounted in the same solution and imaged within a week. Root apices were observed using an optic microscope (Olympus BX61 TRF, Japan) under natural fluorescence conditions using UV illumination (360–370 nm) to allow observation of cell walls in epidermal root cells. Individual root apices (Figure 1) were imaged at 10 x magnification by gathering 2–3 contiguous images, until the zone where root hair development was observed.

All image processing and data extraction were performed manually using the ImageJ image analysis software (Rasband WS. U.S. National Institutes of Health, Bethesda, MD, USA). The contrast of the microscopy images was first adjusted to make the cell files appear as clearly as possible. Cell lengths from all clearly visible files (usually the central 3-4 cell files) of the epidermal tissue were manually measured for each root as the distance between two consecutive transverse cell walls. Cell length sampling started at the root cap junction and spanned shootward, as long as the quality of the image allowed it, and in all cases far beyond the occurrence of the first root hair. Each cell was assigned to a longitudinal position equal to its orthogonal projection onto a virtual line passing through the middle of the root, taking the root cap junction as the origin. The onset of root hair formation was estimated for each root by locating the most rootward cell showing an incipient root hair bulge. Lateral root diameter was evaluated at two positions beyond the first root hair. Two hours were generally sufficient to process a typical root sample. After exploration of cell length profiles, 36 lateral roots were retained for further analyses (18 wild-type, 8 *rtcs* and 10 *rum-1* individuals), the 6 others being rejected because of a too sparse sampling of cells. An average of 160 cell lengths was measured for each selected root, with a minimum of 52 and a maximum of 267.

### Multiple change-point models for identifying development zones in lateral root cell length profiles

#### Definition of heteroscedastic piecewise Gaussian linear models and Gaussian change in the variance models

Multiple change-point models were used to delimit developmental zones within a cell length series **x** of length *T*. We made the assumption of heteroscedastic piecewise Gaussian linear models where the within-zone parameters were the intercept, slope and residual variance. The heteroscedasticity assumption (a residual variance different in each zone) was suggested by the data characteristics and validated *a posteriori*.

We adopted a retrospective or off-line inference approach whose objective was to infer the number of developmental zones *J*, the positions of the *J* − 1 change points τ_1_, …, τ_*J*−1_ (with the convention τ_0_ = 1 and τ_*J*_ = *T* + 1), the *J* within-zone intercepts α_*j*_, slopes β_*j*_ and residual variance $\sigma_{j}^{2}$. For the selection of the number of developmental zones, we used the slope heuristic proposed by Guédon (2015b). The principle of this kind of penalized likelihood criterion consists in making a trade-off between an adequate fitting of the model to the data and a reasonable number of parameters to be estimated.

Once the number of developmental zones *J* had been selected, the cell length series was optimally segmented into *J* zones using a dynamic programming algorithm (Auger and Lawrence, 1989). This optimal segmentation (i.e., the most probable segmentation among all the possible segmentations for a fixed number of developmental zones) defines the optimal change points and relies on the estimation of within-zone parameters. It thus defines the optimal piecewise linear function which is not assumed to be continuous at change points. This optimal piecewise linear function should only be considered as the most likely explanation of the cell length series using multiple change-point models but not as a generative model of the underlying biological mechanism. Guédon (2013) generalized the dynamic programming algorithm of Auger and Lawrence (1989) to compute the top *N* most probable segmentations. This algorithm was useful since, in some cases, a well-supported alternative segmentation was more consistent with biological assumptions than the optimal segmentation.

The assessment of multiple change-point models relied on two posterior probabilities (see Methods S1 in Supplementary Material for formal definitions):

- posterior probability of the selected *J*-developmental-zone model, i.e. weight of the *J*-developmental-zone model among all the possible models. This posterior probability is an output of the slope heuristic (see Methods S1 in Supplementary Material).
- posterior probability of the optimal segmentation in *J* developmental zones, i.e. weight of the optimal segmentation among all the possible segmentations in *J* developmental zones.

These two posterior probabilities reflect the hierarchical nature of the inference with two successive steps: (i) selection of the number of developmental zones using the slope heuristic considering all the possible segmentations in *J* developmental zones for *J* = 1, …, *J*_max_ and (ii) computation of the optimal segmentation in the number of developmental zones previously selected.

We used different diagnostic tools (Guédon, 2013) to assess the assumption of the segmentation in developmental zones and in particular two types of posterior probability profiles that summarize all the possible segmentations for a fixed number of developmental zones: posterior zone probability profiles and posterior segmentation probability profiles. It is often of interest to quantify the uncertainty concerning change-point position. To this end, we computed uncertainty interval for each change point using the smoothing algorithm proposed by Guédon (2013). All these quantities used for diagnostic are formally defined in Methods S1 in Supplementary Material.

We conducted a residual analysis to decipher the weights of the change in slope and of the change in residual variance in the determination of change points. To this end, we computed the residual series by subtracting the piecewise linear function corresponding to the selected segmentation from the measured series. We then estimated a Gaussian change in the variance model applying the methodology previously described for heteroscedastic piecewise Gaussian linear models. In a Gaussian change in the variance model, we suppose that *J* − 1 change points τ_1_ < … < τ_*J*−1_ exist such that the mean is assumed to be constant and the variance is constant between two successive change points:

$$\begin{array}{l} \text{if }\tau_{j}\leq t<\tau_{j+1},\;\{\begin{array}{c} E(X_{t})=\alpha ,\\ \text{Var}(X_{t})=\sigma_{j}^{2}. \end{array} \end{array}$$

In our context of residual analysis, the estimated mean was always very close to 0. Details on the statistical methods for multiple change-point models are given in Methods S1 in Supplementary Material.

#### Illustration of the application of multiple change-point models on selected maize lateral root apices

Three successive zones are expected along the apex of growing roots starting from the tip: the root apical meristem (called division zone and abbreviated DZ hereafter), the elongation zone (EZ) and the mature zone (MZ). We assumed that the division zone was characterized by small cells, the elongation zone by cells of gradually increasing length and the mature zone by large cells. In our modeling framework, the limit between two successive zones corresponds to a marked change in slope and in residual standard deviation.

**Figure 2:**
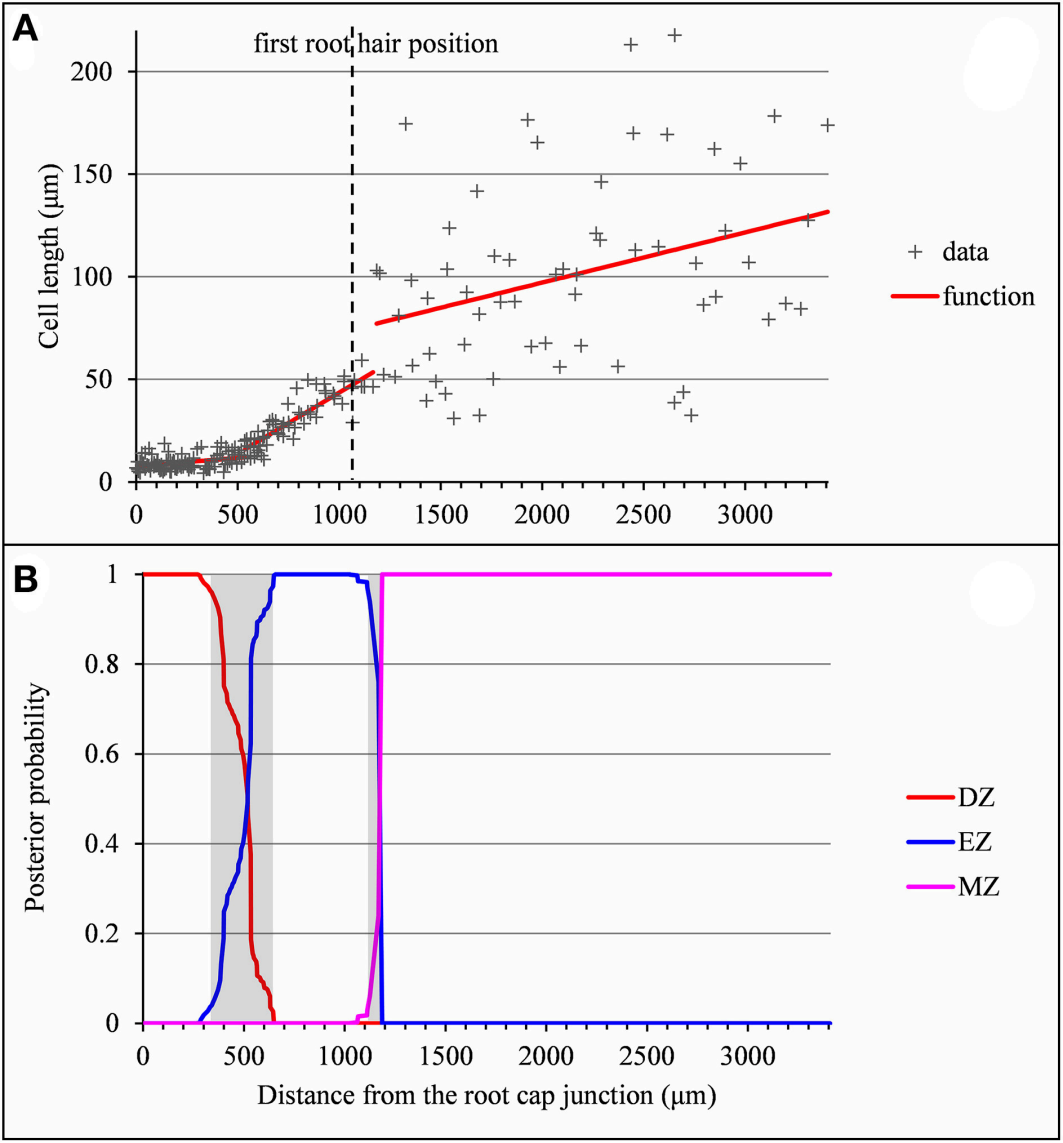
Outputs of the selected piecewise linear model in the case of a typical vigorous lateral root (*rtcs* A1). **(A)** Optimal 3-segment piecewise linear function and first root hair position; **(B)** Posterior division zone (DZ), elongation zone (EZ), and mature zone (MZ) probabilities. The uncertainty intervals for the DZ-EZ and EZ-MZ limits are in gray.

**Figure 3:**
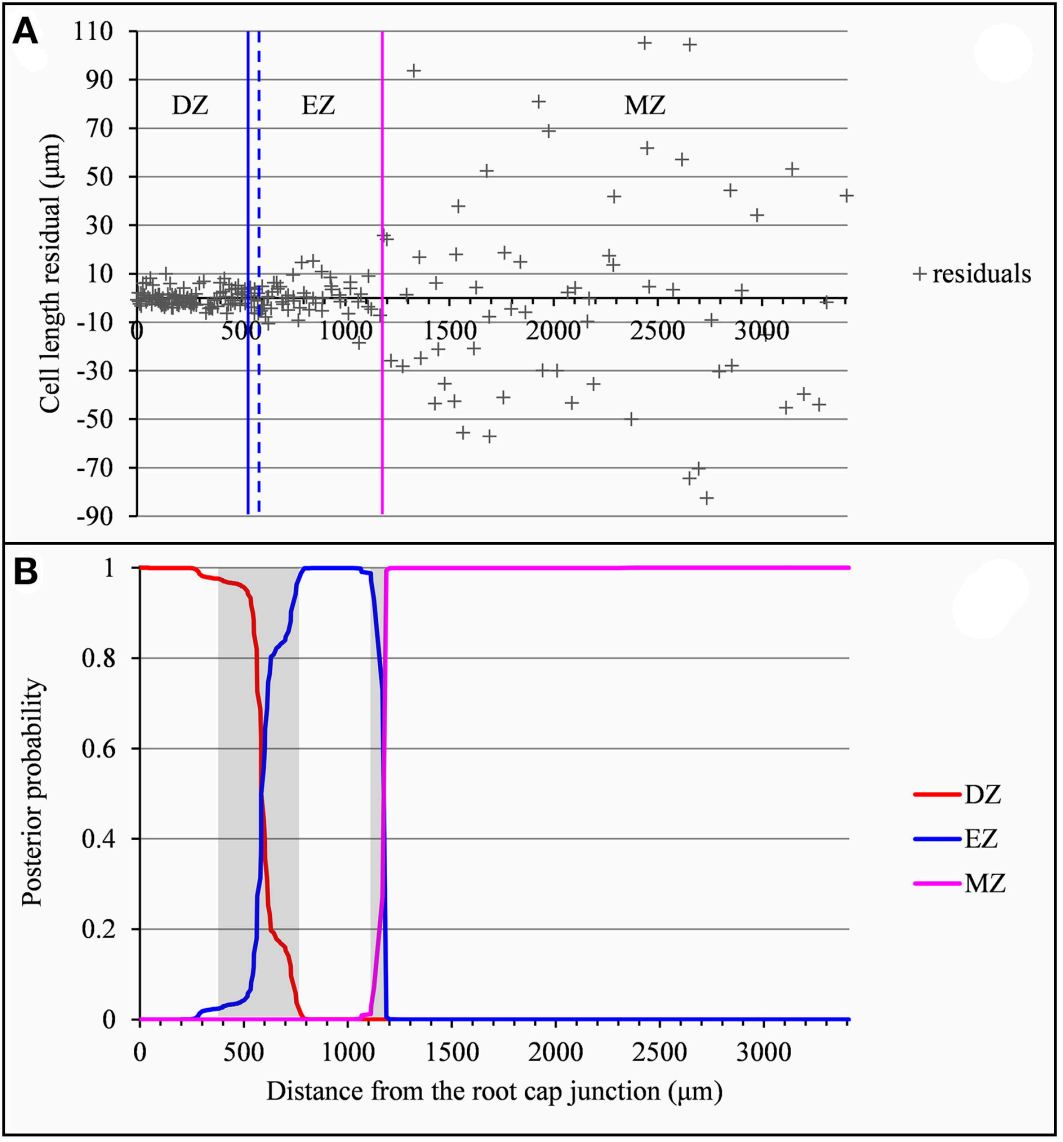
Residual analysis of the lateral root (*rtcs* A1) presented in Figure 2. **(A)** Segmentation in 3 zones of the residual series using a Gaussian change in the variance model with the division zone-elongation zone (DZ-EZ) and elongation zone-mature zone (EZ-MZ) limits (solid lines: estimated on the basis of the original series; dotted lines: estimated on the basis of the residuals series); **(B)** Posterior DZ, EZ, and MZ probabilities. The uncertainty intervals for the DZ-EZ and EZ-MZ limits are in gray.

The example presented in Figure 2 illustrates a typical type A lateral root where the DZ-EZ and EZ-MZ limits correspond to changes both in slope and in residual standard deviation. The residual analysis (Figure 3) highlights the role played by the change in residual standard deviation for defining limits between consecutive zones since the uncertainty intervals for the DZ-EZ and EZ-MZ limits given by the piecewise linear model estimated on the basis of the measured series and the change in the variance model estimated on the basis of the residual series are very close.

**Figure 4:**
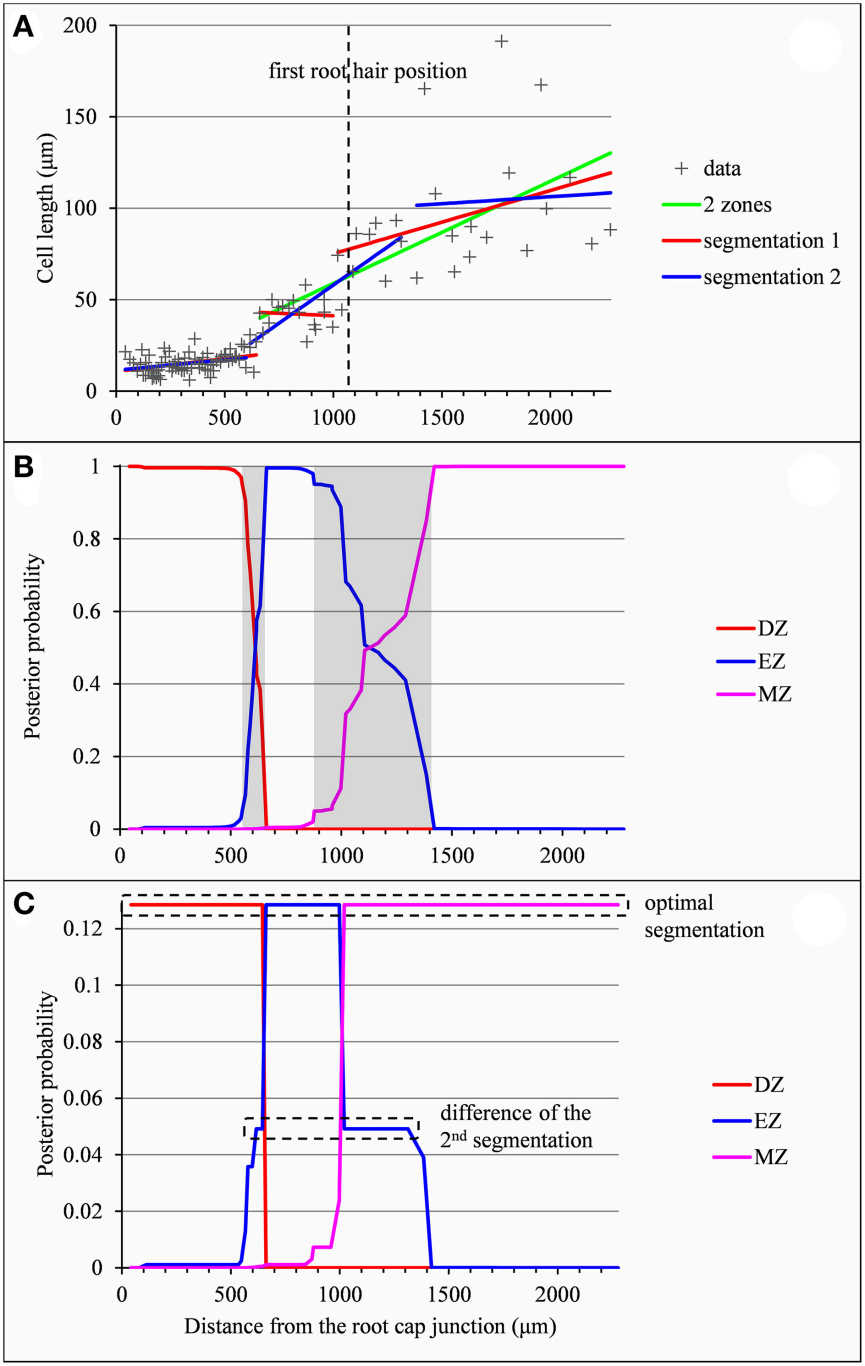
Outputs of the piecewise linear models in the case of a lateral root (*rtcs* A3) for which the 2-zone model selected by the slope heuristic and the optimal 3-segment piecewise linear function do not fit biological assumptions (lack of the elongation zone and null slope within the elongation zone respectively). **(A)** Optimal 2-segment and 3-segment piecewise linear functions, sub-optimal 3-segment piecewise linear function and first root hair position; **(B)** Posterior division zone (DZ), elongation zone (EZ) and mature zone (MZ) probabilities; **(C)** Posterior segmentation probabilities highlighting the difference between the 2nd segmentation and the optimal segmentation in 3 zones.

In a few cases, the optimal piecewise linear function did not fit our biological assumptions. The type A lateral root presented in Figure 4 illustrates such a case where the optimal piecewise linear function deduced from the optimal 2-segment model can be interpreted as a division zone followed by a mature zone according to our biological assumptions. The missing elongation zone could not be identified in the optimal 3-segment piecewise linear function (the slope in the elongation zone is not significantly different from zero) but rather in the well-supported alternative 3-segment piecewise linear function corresponding to the second segmentation. In this example, the difficulty comes from the rather sparse sampling of cells in the mature zone (16 cells in the retained segmentation instead of 62 in the example shown in Figure 2) in conjunction with the high MZ residual standard deviation. As a consequence, the uncertainty interval for the EZ-MZ limit is large. The example presented in Figure S1 illustrates a similar situation but where the optimal 3-segment piecewise linear function is consistent with our biological assumptions.

**Figure 5:**
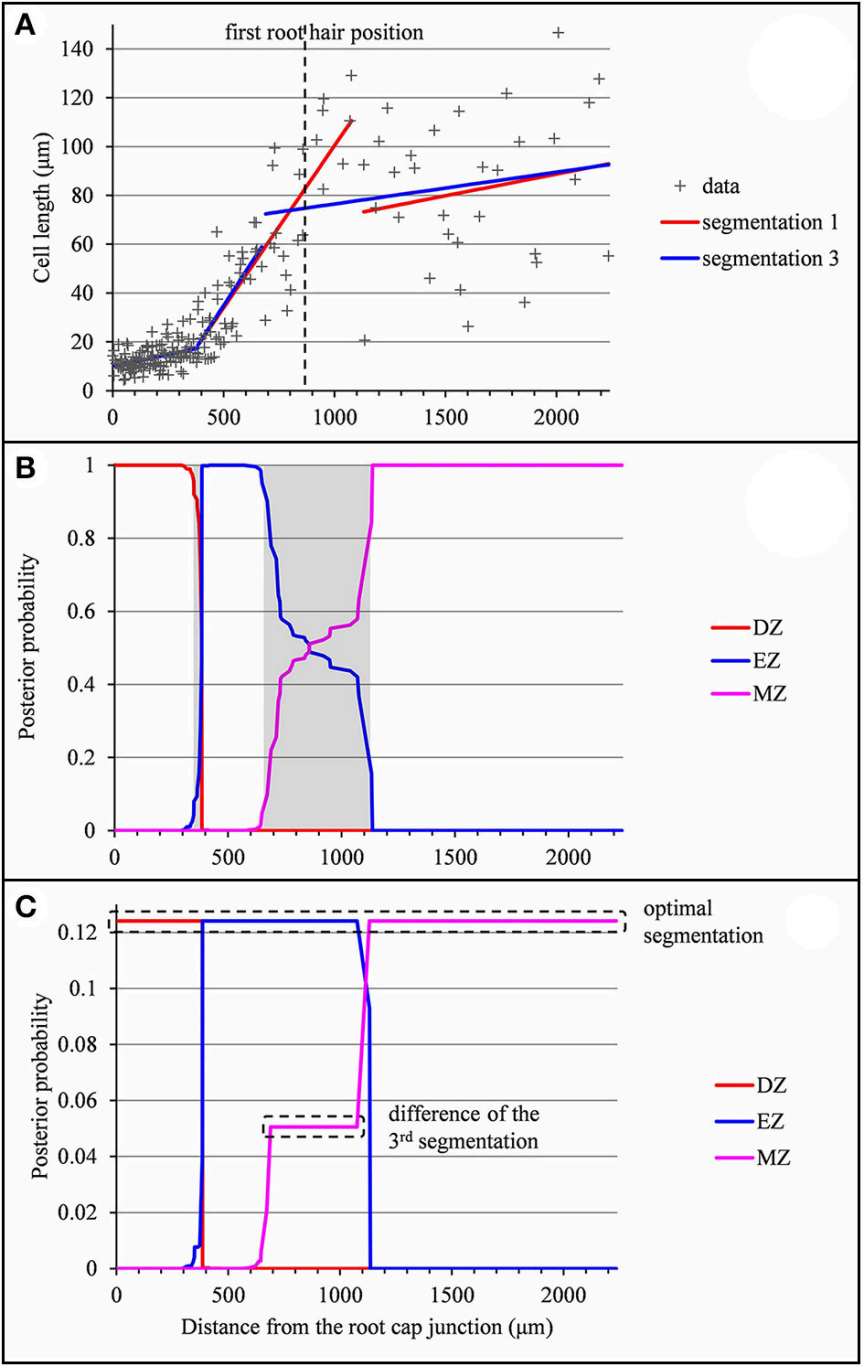
Outputs of the selected piecewise linear model in the case of a lateral root (*rum-1* A′40) for which the optimal 3-segment piecewise linear function does not fit biological assumptions (piecewise linear function not approximately continuous). **(A)** Optimal 3-segment piecewise linear function, sub-optimal 3-segment piecewise linear function corresponding to the 3rd segmentation and first root hair position; **(B)** Posterior division zone (DZ), elongation zone (EZ), and mature zone (MZ) probabilities; The uncertainty intervals for the DZ-EZ and EZ-MZ limits are in gray. **(C)** Posterior segmentation probabilities highlighting the difference between the 3rd segmentation and the optimal segmentation.

The example presented in Figure 5 illustrates the case of a type A lateral root for which the determination of the EZ-MZ limit is rather uncertain. The optimal limit at 1,132 μm is at the shootward end of the uncertainty interval. This limit entails a jump of −44.7 μm between the two linear functions. We thus retained the limit at 689 μm corresponding to the third segmentation which only entails a jump of 11.1 μm. It is also consistent with the residual analysis since the optimal segmentation of the residual series deduced either from the optimal segmentation or from the third segmentation has the same limits as the third segmentation of the measured series. The example presented in Figure S2 illustrates a similar situation in the case of a type B lateral root without a division zone for which the second segmentation is far more consistent with the approximate continuity of the selected piecewise linear function.

These examples illustrate the strategy we adopted for selecting piecewise linear functions combining the inference of multiple change-point models with biological assumptions. We first computed the optimal piecewise linear function for the number of developmental zones given by the slope heuristic. We then identified the division zone, the elongation zone and the mature zone and checked their characteristics according to our biological assumptions (knowing that the division zone or the division and the elongation zones can be absent in type B or C roots). If two consecutive zones were merged (e.g., the elongation and the mature zones for the example presented in Figure 4), we explored the well-supported (in terms of posterior probabilities) segmentations with one more zone. If the optimal piecewise linear function was strongly inconsistent regarding the approximate continuity assumption (the presence of a huge discontinuity was rather inconsistent with the biological assumption of a gradual increase in cell length), we explored well-supported alternative segmentations. No other biological assumptions (e.g., position of the EZ-MZ limit with respect to the first root hair position) were used for selecting piecewise linear functions.

## Results

We assumed that the developmental pattern was common to the studied lateral roots even if the most rootward developmental zones (i.e., the division zone or the division and the elongation zones) were absent in some roots. The analysis of this developmental pattern broke down in two steps:

1. Identification and characterization of the successive developmental zones along each lateral root. For this individual analysis, we focused in particular on the selection of the number of developmental zones, on the roles played by the change in slope and the change in residual standard deviation in the determination of the limits between these zones and on the uncertainty concerning these limits.
2. Comparison of the developmental zones of the lateral roots in order to identify commonalities and differences between these zonations.

### Selection of the number of developmental zones

**Figure 6:**
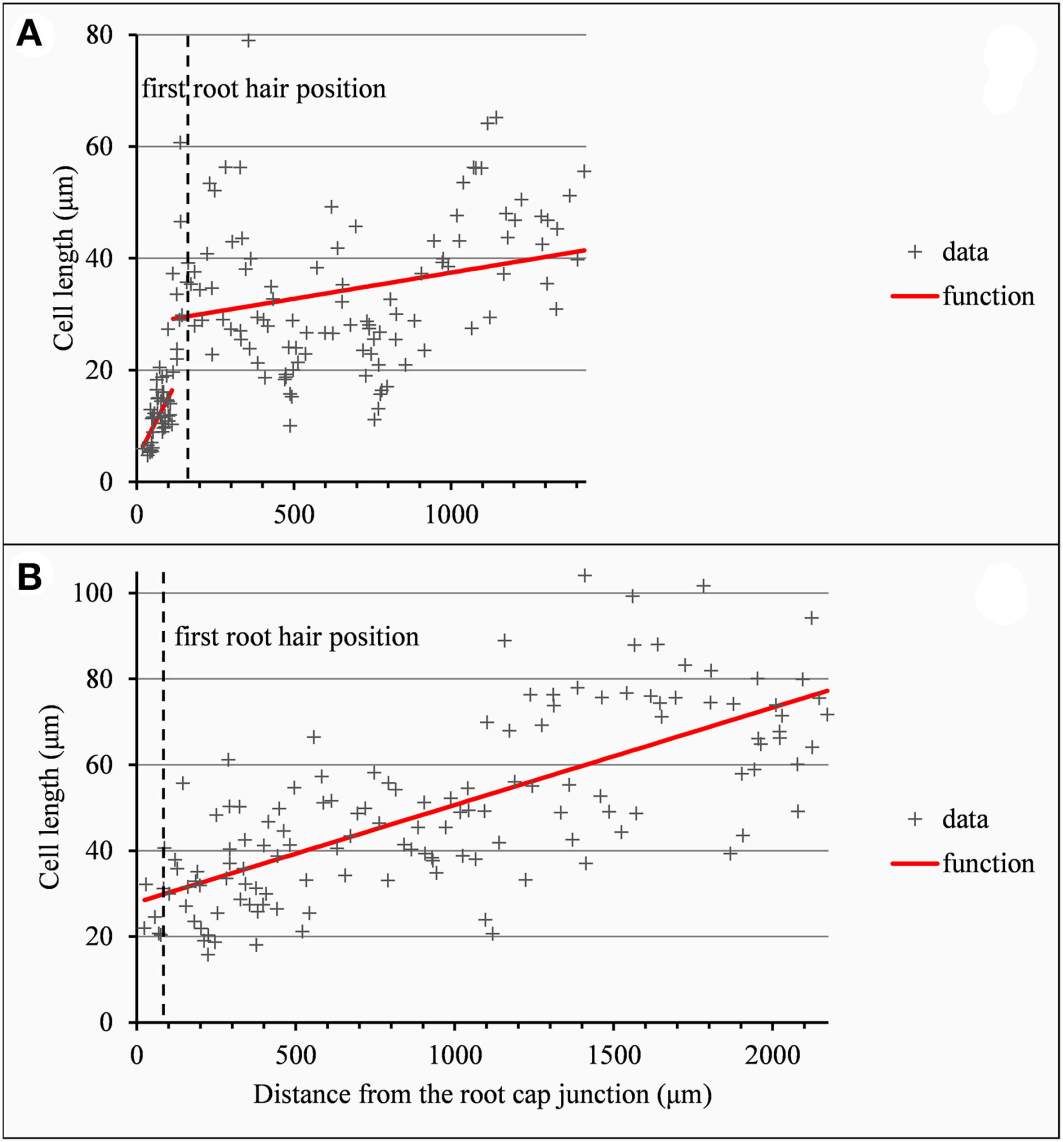
Outputs of the selected piecewise linear model in the case of 2-zone −elongation zone (EZ) and mature zone (MZ)− individual and a single-zone −mature zone only− individual corresponding to arrested or almost arrested roots. **(A)** Wild-type C28: optimal 2-segment piecewise linear function and first root hair position; **(B)** Wild-type C27: optimal linear function and first root hair position.

We retained the number of developmental zones given by the slope heuristic and the optimal segmentation in this number of developmental zones for 26 individuals among 36 (Tables 1–3 for the wild type and the *rtcs* and *rum-1* mutant respectively). This includes the three 4-zone individuals presented in Tables S1, S2 (see below for the interpretation of this pattern). Figure 2 shows a typical 3-zone individual while Figure 6 shows a typical 2-zone (elongation and mature zones) individual and a typical single-zone (mature zone only) individual both categorized as type C and illustrating the diversity of cell length pattern in arrested roots. For two individuals, we retained a well-supported alternative model with one more developmental zone than the model selected by the slope heuristic and for two other individuals (see Figure 5, Figure S2), we retained a well-supported alternative segmentation in the number of developmental zones given by the slope heuristic. For the 6 remaining individuals, we retained a model with one more developmental zone than the model selected by the slope heuristic and the optimal segmentation in this number of developmental zones (see Figure S1) except for one individual (see Figure 4) for which we retained a well-supported alternative segmentation. All these choices of alternative model or segmentation were supported by the biological assumptions stated before regarding the succession of developmental zones and their main properties in terms of cell length. It should be noted that among the 10 individuals for which we did not retain the number of developmental zones given by the slope heuristic and the optimal segmentation in this number of developmental zones, 7 were characterized by a rather sparse sampling of cells within the mature zone (wild-type A8, *rtcs* A3, A2, A′39, B15, *rum-1* A7, A′40); see Tables 4–6.

**Table 1:** Multiple change-point models estimated for wild-type lateral roots.

								**Posterior probability**		
		**DZ s.d**.	**DZ-EZ limit**	**EZ s.d**.	**EZ-MZ limit**	**First hair position**	**MZ s.d**.	**Segmentation**	**Model**	**SH model**
A10	Linear	1.3	855 (761, 855)	5.5	1,494 (1,121, 1,556)	2,122	18.2	0.16^*^	1^*^	3
	Variance	1.3	855 (766, 867)	5.5	1,494 (1,188, 1,561)		18.1	0.17^*^	0.54^*^	3
A31	Linear	2.2	676 (633, 763)	4.6	1,368 (1,323, 1,368)	1,692	29.9	0.1^*^	1^*^	3
	Variance	2.6	988 (898, 1,019)	5.8	1,368 (1,323, 1,370)		29.7	0.1^*^	0.95^*^	3
A9	Linear	2	587 (528, 635)	3.5	1,219 (1,177, 1,242)	2,318	27.8	0.13^*^	1^*^	3
	Variance	2.2	1,008 (973, 1,053)	7.3	1,531 (1,440, 2,295)		30.4	0.07^*^	0.95^*^	3
A8	Linear	0.8	553 (532, 553)	5.4	1,100 (1,001, 1,100)	1,862	17	0.13^*^	0.29	2
	Variance	0.7	553 (532, 553)	4.4	1,030 (980, 1,100)		15.9	0.16^*^	0.98^*^	3
A13	Linear	2	510 (459, 521)	6	973 (952, 973)	1,267	37.5	0.24^*^	0	4
	Variance	2	511 (489, 521)	6	1,013 (915, 1,013)		37.5	0.25^*^	0.91^*^	3
B33	Linear	1.8	439 (387, 439)	8.1	672 (631, 672)	781	33.4	0.21^*^	0	4
	Variance	1.8	458 (427, 473)	8.4	672 (628, 672)		33.3	0.21^*^	0.98^*^	3
B32	Linear	2.2	411 (337, 411)	6.7	716 (672, 716)	929	24.7	0.35^*^	0	4
	Variance	2.2	411 (337, 517)	6.8	719 (672, 824)		24.9	0.1^*^	0.02	4
B19	Linear	1.7	366 (344, 396)	4.9	600 (578, 770)	895	32.5	0.17^*^	1^*^	3
	Variance	1.8	415 (358, 435)	5.3	600 (568, 600)		32.1	0.21^*^	0.98^*^	3
A11	Linear	1.9	328 (255, 383)	3.6	655 (647, 655)	1,165	24.1	0.13^*^	0.96^*^	3
	Variance	2.2	454 (277, 474)	4.6	678 (654, 678)		24.3	0.1^*^	0.98^*^	3
A12	Linear	1.7	320 (314, 320)	5.1	722 (677, 722)	756	25.6	0.5^*^	1^*^	3
	Variance	1.7	320 (314, 363)	5.2	729 (677, 729)		25.6	0.26^*^	1^*^	3
B34	Linear	1.2	278 (274, 318)	6.3	461 (392, 461)	515	24.6	0.27^*^	0.85^*^	3
	Variance	1.2	278 (270, 297)	4	392 (391, 474)		22.2	0.17^*^	0.13	4
B20	Linear	2.7	232 (227, 253)	10.1	574 (517, 696)	586	25.3	0.14^*^	1^*^	3
	Variance	2.7	232 (224, 253)	9.8	591 (349, 591)		25.7	0.2^*^	0.91^*^	3
B35	Linear	3	215 (171, 224)	7.2	388 (367, 446)	430	23.2	0.19^*^	1^*^	3
	Variance	3.3	257 (207, 293)	12	1,157 (388, 1,157)		29.9	0.02^*^	0.72^*^	3
C25	Linear			3.2	144 (67, 180)	121	11.6	0.37^*^	0.01	1
	Variance			3.1	144 (67, 722)		11.6	0.44^*^	0.9^*^	2
C28	Linear			4.2	115 (106, 115)	166	12.8	0.65^*^	1^*^	2
	Variance			4.5	139 (97, 139)		13	0.21^*^	1^*^	2
C26	Linear					101	11.5	1^*^	1^*^	1
	Variance						11.5	1^*^	0.27	2
C27	Linear					93	14	1^*^	1^*^	1
	Variance						14	1^*^	0.61^*^	1
C30	Linear					197	15.9	1^*^	1^*^	1
	Variance						15.9	1^*^	0.95^*^	1

*For each lateral root (ordered in decreasing length of the division zone), the piecewise linear model is described in the first row and the Gaussian change in the variance model estimated on the basis of the residual series is described in the second row. For each multiple change-point model, the standard deviations (s.d.) estimated for each zone −division zone (DZ), elongation zone (EZ) and mature zone (MZ)−, the limits between zones with associated 0.05-uncertainty intervals, the first root hair position (all in μm), the selected segmentation posterior probability −an asterisk indicates that the segmentation is the optimal one−, the selected model posterior probability −an asterisk indicates that the model is the one given by the slope heuristic (SH)−, and the number of zones given by the slope heuristic are given*.

**Table 2:** Multiple change-point models estimated for *rtcs* lateral roots.

								**Posterior probability**		
		**DZ s.d**.	**DZ-EZ limit**	**EZ s.d**.	**EZ-MZ limit**	**First hair position**	**MZ s.d**.	**Segmentation**	**Model**	**SH model**
A3	Linear	4.8	617 (548, 662)	12.9	1,385 (879, 1,421)	1,083	40.1	0.05	0	2
	Variance	4.7	634 (553, 718)	13.1	1,385 (879, 1,421)		38.7	0.19^*^	0.53^*^	3
A1	Linear	3.4	535 (321, 649)	6	1,185 (1,112, 1,185)	1,078	43.6	0.14^*^	1^*^	3
	Variance	3.4	583 (387, 775)	6.3	1,185 (1,112, 1,185)		43.3	0.08^*^	0.96^*^	3
A′36	Linear	2.1	506 (474, 506)	6.6	1,146 (997, 1,215)	965	21.8	0.13^*^	0.61^*^	3
	Variance	2.1	506 (465, 510)	6.5	1,146 (997, 1,241)		21.3	0.15^*^	0.99^*^	3
A2	Linear	3.1	323 (16, 347)	11.4	744 (388, 828)	485	28	0.13^*^	0.01	2
	Variance	3.1	347 (68, 347)	11.8	744 (347, 796)		27.6	0.12^*^	0.17	2
A′37	Linear	1.3	313 (298, 313)	6.1	707 (549, 828)	692	14.7	0.1^*^	0.95^*^	3
	Variance	1.3	313 (288, 313)	3.8	455 (417, 707)		12.3	0.08^*^	0.94^*^	3
A′38	Linear	1.4	272 (267, 322)	4.3	505 (426, 505)	563	16.1	0.05^*^	0	2
	Variance	1.5	295 (285, 318)	4.3	428 (402, 697)		15.5	0.05^*^	0.88^*^	3
A′39	Linear			8.8	980 (548, 1,118)	634	26.9	0.35^*^	0.2	1
	Variance			8.6	980 (548, 1,118)		26.4	0.39^*^	0.91^*^	2
B15	Linear			5.8	520 (328, 819)	210	15.2	0.05	1^*^	2
	Variance			5.6	596 (101, 654)		15.6	0.36^*^	0.9^*^	2

*For each lateral root (ordered in decreasing length of the division zone), the piecewise linear model is described in the first row and the Gaussian change in the variance model estimated on the basis of the residual series is described in the second row. For each multiple change-point model, the standard deviations (s.d.) estimated for each zone −division zone (DZ), elongation zone (EZ) and mature zone (MZ)−, the limits between zones with associated 0.05-uncertainty intervals, the first root hair position (all in μm), the selected segmentation posterior probability −an asterisk indicates that the segmentation is the optimal one−, the selected model posterior probability −an asterisk indicates that the model is the one given by the slope heuristic (SH)−, and the number of zones given by the slope heuristic are given*.

**Table 3:** Multiple change-point models estimated for *rum-1* lateral roots.

								**Posterior probability**		
		**DZ s.d**.	**DZ-EZ limit**	**EZ s.d**.	**EZ-MZ limit**	**First hair position**	**MZ s.d**.	**Segmentation**	**Model**	**SH model**
A5	Linear	2.5	787 (771, 820)	8.8	2,360 (2,090, 2,360)	2,032	51.7	0.38^*^	1^*^	3
	Variance	2.6	842 (787, 858)	9.2	2,360 (2,185, 2,360)		50.8	0.41^*^	1^*^	3
A7	Linear	1.8	456 (415, 491)	7	1,123 (610, 1,123)	1,241	19.4	0.1^*^	0	2
	Variance	1.8	456 (393, 463)	4.1	629 (595, 1,050)		12.3	0.03^*^	0.97^*^	3
A′41	Linear	4.6	452 (392, 469)	9.8	1,246 (787, 1,352)	1,023	33.2	0.06^*^	0.74^*^	3
	Variance	4.5	452 (401, 482)	9.6	1,352 (1,107, 1,433)		34.3	0.12^*^	0.99^*^	3
A4	Linear	2.1	399 (379, 445)	6.1	1,068 (941, 1,187)	869	18.6	0.1^*^	0.95^*^	3
	Variance	2.4	542 (507, 542)	7	1,187 (1,103, 1,187)		19.8	0.31^*^	0.99^*^	3
A′40	Linear	4.4	385 (343, 385)	12	689 (647, 1,136)	885	30	0.05	0.97^*^	3
	Variance	4.4	385 (310, 385)	11.8	689 (639, 770)		29.7	0.17^*^	0.97^*^	3
A6	Linear	2.1	371 (347, 451)	4.7	958 (846, 958)	1,700	29.4	0.25^*^	1^*^	3
	Variance	2.1	371 (347, 547)	4.7	958 (909, 958)		28.9	0.28^*^	0.94^*^	3
A′42	Linear	3.2	295 (178, 352)	5.5	627 (499, 627)	585	20.7	0.14^*^	0.93^*^	3
	Variance	2.8	225 (140, 297)	5.2	627 (548, 627)		20.5	0.07^*^	0.29	2
C22	Linear			5.4	1,510 (1,289, 1,510)	1,270	15.2	0.85^*^	1^*^	2
	Variance			5.4	1,867 (1,752, 1,897)		17.5	0.33^*^	1^*^	2
C24	Linear			4	540 (482, 637)	656	11.6	0.5^*^	0.94^*^	2
	Variance			4	540 (482, 637)		11.5	0.46^*^	0.91^*^	2
C23	Linear			7.8	732 (456, 909)	421	10.3	0.15^*^	0	1
	Variance						9.1	1^*^	0.99^*^	1

*For each lateral root (ordered in decreasing length of the division zone), the piecewise linear model is described in the first row and the Gaussian change in the variance model estimated on the basis of the residual series is described in the second row. For each multiple change-point model, the standard deviation (s.d.) estimated for each zone −division zone (DZ), elongation zone (EZ) and mature zone (MZ)−, the limits between zones with associated 0.05-uncertainty intervals, the first root hair position (all in μm), the selected segmentation posterior probability −an asterisk indicates that the segmentation is the optimal one−, the selected model posterior probability −an asterisk indicates that the model is the one given by the slope heuristic (SH)−, and the number of zones given by the slope heuristic are given*.

**Table 4:** Piecewise linear functions selected for wild-type lateral roots.

	**Division zone**				**Elongation zone**				**Mature zone**			
	**Slope**	**Correlation**	**No. cells**	**DZ-EZ limit**	**Slope**	**Correlation**	**No. cells**	**EZ-MZ limit**	**Slope**	**Correlation**	**No. cells**	**End position**
A10	0.8	0.12 n.s.	132	855	59.5	0.88	67	1,494	49.6	0.78	68	2,990
A31	−6	−0.42	78	676	50.5	0.9	95	1,368	65.9	0.73	70	3,126
A9	−5.3	−0.35	94	587	42.4	0.91	61	1,219	43.1	0.76	67	3,557
A8	−3.2	−0.36 n.s.	17	553	59.2	0.83	34	1,100	49.4	0.77	37	2,459
A13	−3.6	−0.24	109	510	90.9	0.87	76	973	30.1	0.35	53	2,582
B33	−0.2	−0.01 n.s.	103	439	201.8	0.82	36	672	15.1	0.21 n.s.	59	2,198
B32	−16	−0.53	110	411	116.3	0.81	48	716	35.2	0.44	39	1,852
B19	11.6	0.42	30	366	118.8	0.85	31	600	21.2	0.36	36	2,283
A11	−6.3	−0.25 n.s.	55	328	83	0.9	77	655	74.6	0.72	50	1,898
A12	1.3	0.05 n.s.	49	320	102.5	0.91	44	722	13.3	0.33	77	2,967
B34	−9.6	−0.4	27	278	140.1	0.72	57	461	27.6	0.55	68	2,182
B20	1	0.02 n.s.	48	232	201.4	0.88	52	574	15.9	0.44	34	2,775
B35	−22.1	−0.32	44	215	183.3	0.76	44	388	3.2	0.09 n.s.	79	2,771
C25					159	0.75	18	144	30.6	0.82	110	1,961
C28					108.6	0.53	40	115	9.4	0.26	111	1,424
C26									28.1	0.82	133	2,115
C27									22.7	0.72	136	2,172
C30									27.6	0.69	170	2,214

*For each lateral root (ordered in decreasing length of the division zone), the parameters of the piecewise linear function (slope × 1,000 in μm/mm, correlation coefficient for each zone –division zone (DZ), elongation zone (EZ) and mature zone (MZ), n.s. for non-significant−, limits between zones and position of the most shootward cell in μm) and the number of cells assigned to each zone are given*.

**Table 5:** Piecewise linear functions selected for *rtcs* lateral roots.

	**Division zone**				**Elongation zone**				**Mature zone**			
	**Slope**	**Correlation**	**No. cells**	**DZ-EZ limit**	**Slope**	**Correlation**	**No. cells**	**EZ-MZ limit**	**Slope**	**Correlation**	**No. cells**	**End position**
A3	11.2	0.34	67	617	83.4	0.81	29	1,385	7.7	0.05 n.s.	16	2,277
A1	8.3	0.36	92	535	59.6	0.88	60	1,185	24.5	0.34	62	3,407
A′36	2.3	0.15 n.s.	116	506	92.2	0.93	39	1,146	25.5	0.3 n.s.	22	1,998
A2	27.9	0.64	68	323	157.5	0.86	29	744	10.1	0.17 n.s.	35	2,257
A′37	20.4	0.63	42	313	82.2	0.84	51	707	31.2	0.47	40	1,514
A′38	9.7	0.51	46	272	111.7	0.82	41	505	28.7	0.49	47	1,621
A′39					66	0.84	37	980	23.5	0.23 n.s.	27	1,844
B15					139.5	0.96	30	520	-9.5	−0.18 n.s.	22	1,493

*For each lateral root (ordered in decreasing length of the division zone), the parameters of the piecewise linear function (slope × 1,000 in μm/mm, correlation coefficient for each zone –division zone (DZ), elongation zone (EZ), and mature zone (MZ), n.s. for non-significant−, limits between zones and position of the most shootward cell in μm) and the number of cells assigned to each zone are given*.

**Table 6:** Piecewise linear functions selected for *rum-1* lateral roots.

	**Division zone**				**Elongation zone**				**Mature zone**			
	**Slope**	**Correlation**	**No. cells**	**DZ-EZ limit**	**Slope**	**Correlation**	**No. cells**	**EZ-MZ limit**	**Slope**	**Correlation**	**No. cells**	**End position**
A5	−3.3	−0.28	157	787	65	0.96	73	2,360	21.1	0.22 n.s.	29	4,090
A7	3	0.15 n.s.	71	456	62	0.85	55	1,123	41	0.58	13	2,109
A′41	−6	−0.17 n.s.	75	452	75	0.84	61	1,246	16.9	0.18 n.s.	16	2,329
A4	0	0 n.s.	101	399	42.3	0.8	87	1,068	6.5	0.08 n.s.	46	1,870
A′40	19.5	0.43	122	385	139.9	0.71	41	689	13	0.2 n.s.	52	2,235
A6	−14.7	−0.57	68	371	43.1	0.83	67	958	73.7	0.64	31	2,139
A′42	26.7	0.57	73	295	145.3	0.93	33	627	2.4	0.06 n.s.	55	2,325
C22					11.6	0.61	73	1,510	14.4	0.34	38	2,820
C24					9.8	0.38	62	540	13.5	0.67	81	3,247
C23					85.2	0.85	39	732	22.6	0.64	48	2,008

*For each lateral root (ordered in decreasing length of the division zone), the parameters of the piecewise linear function (slope × 1,000 in μm/mm, correlation coefficient for each zone –division zone (DZ), elongation zone (EZ) and mature zone (MZ), n.s. for non-significant−, limits between zones and position of the most shootward cell in μm) and the number of cells assigned to each zone are given*.

For three wild-type individuals, 4 zones were identified where the first two zones correspond to a split of the division zone (Table 1, Tables S1, S2) that can be interpreted as a proliferation zone followed by a transition zone. The segmentations in 3 and 4 zones are nested or almost nested in the case of A13 (Figure S3). The limit between the two successive zones within the division zone corresponded mainly to a change in slope with a negative slope in the first zone followed by a positive slope or a slope non-significantly different from zero in the case of B32 in the second zone (Tables S1, S2). When the residual series was extracted using the 4-segment piecewise linear function, the first two zones could only be identified in B32 but not in the two other individuals for which they were merged consistently with the similar residual standard deviations estimated for these two zones in A13 and B33 (Table S1).

### Approximate continuity of the selected piecewise linear functions

Contrary to segmented regression models (Muggeo, 2003), the piecewise linear functions are not constrained to be continuous in the framework of multiple change-point models. We thus computed the rootward and shootward confidence intervals at each limit between two consecutive developmental zones (e.g., DZ and EZ confidence intervals at the DZ-EZ limit) in order to assess the approximate continuity of the piecewise linear function selected for each individual. The piecewise linear functions are most often approximately continuous for the mutants with overlap between confidence intervals for 13 limits among 14 for *rtcs* (Table 8) and for 15 limits among 17 for *rum-1* (Table 9). The situation is substantially different for the wild type with overlap between confidence intervals for 15 limits among 28, the non-overlap concerning mostly EZ-MZ limits (Table 7).

**Table 7:** Piecewise linear functions selected for wild-type lateral roots.

	**Division zone**			**Elongation zone**			**Mature zone**	
	**Linear function**	**Confidence intervals**	**DZ-EZ limit**	**Linear function**	**Confidence intervals**	**EZ-MZ limit**	**Linear function**	**End position**
A10	5.4 → 6	(5.5, 6.5 | 5, 9.7)	855	7.3 → 45.4	(41.8, 48.9 | 45.7, 61)	1,494	53.3 → 127.6	2,990
A31	9.3 → 5.7	(4.8, 6.7 | 4.2, 8)	676	6.1 → 41	(38.9, 43.2 | 47.7, 70.9)	1,368	59.3 → 175.2	3,126
A9	7.8 → 5.1	(4.2, 6 | 5, 8.3)	587	6.7 → 33.5	(31.5, 35.4 | 40.2, 65)	1,219	52.6 → 153.4	3,557
A8	7.7 → 6.7	(6.1, 7.2 | 2.4, 12.8)	553	7.6 → 40	(36.4, 43.6 | 42.7, 60.7)	1,100	51.7 → 118.8	2,459
A13	7.3 → 5.4	(4.7, 6.2 | 3.9, 9.2)	510	6.5 → 48.6	(45.1, 52.2 | 69.2, 108)	973	88.6 → 137	2,582
B33	6.4 → 6.3	(5.5, 7 | 4.4, 14.8)	439	9.6 → 56.6	(49.2, 64 | 68.6, 99.3)	672	83.9 → 107.1	2,198
B32	9.3 → 3.6	(2.5, 4.7 | 6.1, 13.8)	411	10 → 45.5	(40.7, 50.2 | 60.6, 87.1)	716	73.8 → 113.8	1,852
B19	6.5 → 8.9	(7.8, 9.9 | 7.7, 14.6)	366	11.2 → 39	(34.9, 43 | 66.6, 107.8)	600	87.2 → 122.9	2,283
A11	7.3 → 5.5	(4.3, 6.7 | 3.6, 6.6)	328	5.1 → 32.3	(30.3, 34.2 | 44.1, 65.7)	655	54.9 → 147.6	1,898
A12	5.9 → 6.2	(5.3, 7.1 | 4.5, 9.7)	320	7.1 → 48.3	(44.2, 52.4 | 59, 79.2)	722	69.1 → 98.9	2,967
B34	6.4 → 4.9	(4.1, 5.7 | 1.4, 8.3)	278	4.8 → 30.5	(26.5, 34.5 | 52.7, 72.2)	461	62.4 → 109.9	2,182
B20	11.3 → 11.5	(10, 13 | 6.5, 16.6)	232	11.6 → 80.4	(73.5, 87.4 | 88.5, 115.9)	574	102.2 → 137.2	2,775
B35	9.2 → 5.1	(3.3, 7 | 4.8, 13.2)	215	9 → 40.7	(35.5, 45.9 | 55.1, 71.9)	388	63.5 → 71	2,771
C25				5.2 → 25.7	(18.2, 33.1 | 20.9, 28.4)	144	24.6 → 80.3	1,961
C28				6.3 → 16.7	(13.9, 19.5 | 25, 33.4)	115	29.2 → 41.4	1,424
C26							20.3 → 79.2	2,115
C27							28.5 → 77.2	2,172
C30							28.7 → 89	2,214

*For each lateral root (ordered in decreasing length of the division zone), the piecewise linear function (cell lengths predicted at both ends of a zone –division zone (DZ), elongation zone (EZ) and mature zone (MZ)− linked by an arrow, limits between zones and position of the most shootward cell in μm) with associated rootward and shootward confidence intervals at each limit between zones (between brackets) is given*.

**Table 8:** Piecewise linear functions selected for *rtcs* lateral roots.

	**Division zone**			**Elongation zone**			**Mature zone**	
	**Linear function**	**Confidence intervals**	**DZ-EZ limit**	**Linear function**	**Confidence intervals**	**EZ-MZ limit**	**Linear function**	**End position**
A3	11.9 → 18.3	(15.7, 20.9 | 17.7, 34.4)	617	26 → 90.1	(77.7, 102.4 | 63.7, 139.4)	1,385	101.5 → 108.4	2,277
A1	7.6 → 12	(10.5, 13.6 | 13.3, 18.5)	535	15.9 → 54.6	(50.8, 58.4 | 57.1, 97.3)	1,185	77.2 → 131.6	3,407
A′36	7.5 → 8.6	(7.8, 9.4 | 7.1, 13.8)	506	10.4 → 69.4	(63.8, 75 | 51.1, 86.2)	1,146	68.6 → 90.3	1,998
A2	5.6 → 14.6	(13, 16.2 | 10.4, 24.7)	323	17.5 → 83.8	(73.4, 94.3 | 64.3, 99.5)	744	81.9 → 97.2	2,257
A′37	5.6 → 9.1	(8.3, 9.9 | 7.1, 12.8)	313	10 → 42.4	(38.2, 46.5 | 41.6, 59.2)	707	50.4 → 75.5	1,514
A′38	6.3 → 9	(8.3, 9.6 | 2.7, 6.8)	272	4.8 → 30.8	(26.3, 35.3 | 34.2, 51.7)	505	42.9 → 75	1,621
A′39				11.1 → 59.7	(53.1, 66.3 | 34.3, 74.4)	980	54.4 → 74.7	1,844
B15				8.9 → 79.3	(73.7, 85 | 54.9, 82)	520	68.5 → 59.2	1,493

*For each lateral root (ordered in decreasing length of the division zone), the piecewise linear function (cell lengths predicted at both ends of a zone –division zone (DZ), elongation zone (EZ), and mature zone (MZ)– linked by an arrow, limits between zones, and position of the most shootward cell in μm) with associated rootward and shootward confidence intervals at each limit between zones (between brackets) is given*.

**Table 9:** Piecewise linear functions selected for *rum-1* lateral roots.

	**Division zone**			**Elongation zone**			**Mature zone**	
	**Linear function**	**Confidence intervals**	**DZ-EZ limit**	**Linear function**	**Confidence intervals**	**EZ-MZ limit**	**Linear function**	**End position**
A5	10.2 → 7.7	(6.9, 8.5 | 6.6, 12.9)	787	9.8 → 112	(106.5, 117.6 | 69, 140)	2,360	104.5 → 140.9	4,090
A7	6.7 → 7.8	(6.9, 8.6 | 4.3, 10.5)	456	7.4 → 48.8	(43.7, 53.8 | 51.7, 91.7)	1,123	71.7 → 112.2	2,109
A′41	13.7 → 11	(9.1, 12.9 | 8.4, 16.6)	452	12.5 → 72.1	(64.9, 79.2 | 27.6, 91.6)	1,246	59.6 → 78	2,329
A4	8.9 → 8.9	(8.1, 9.8 | 8.7, 13.1)	399	10.9 → 39.2	(36.1, 42.3 | 38.5, 61)	1,068	49.8 → 55	1,870
A′40	9.9 → 17.4	(15.7, 19.1 | 12.2, 25.4)	385	18.8 → 61.3	(52.1, 70.6 | 58.4, 86.4)	689	72.4 → 92.6	2,235
A6	12 → 6.6	(5.6, 7.6 | 4.4, 8.4)	371	6.4 → 31.6	(28.8, 34.5 | 20.1, 59)	958	39.5 → 126.5	2,139
A′42	7.3 → 15.2	(13.5, 16.9 | 8.9, 15.3)	295	12.1 → 60.3	(55.4, 65.3 | 51.1, 72.1)	627	61.6 → 65.7	2,325
C22				13.2 → 29.2	(26.6, 31.9 | 45.5, 65)	1,510	55.2 → 74.1	2,820
C24				15.4 → 20.7	(18.7, 22.7 | 19.5, 28.4)	540	23.9 → 60.6	3,247
C23				8 → 53.5	(47.1, 59.8 | 34.1, 45.5)	732	39.8 → 68.7	2,008

*For each lateral root (ordered in decreasing length of the division zone), the piecewise linear function (cell lengths predicted at both ends of a zone –division zone (DZ), elongation zone (EZ) and mature zone (MZ)− linked by an arrow, limits between zones and position of the most shootward cell in μm) with associated rootward and shootward confidence intervals at each limit between zones (between brackets) is given*.

### The limits between developmental zones are explained both by a change in slope and in residual standard deviation

We conducted a residual analysis using the residual series deduced from the selected piecewise linear function for each individual. We checked that the residual series were stationary and selected for each series a Gaussian change in the variance model using the slope heuristic (Tables 1–3). We found the same number of zones as for the measured cell length series for 31 individuals among 36 while this number of zones corresponds to a well-supported alternative model for 4 other individuals. Fifty four limits between zones among 59 are co-localized i.e., the uncertainty intervals for a given limit for the piecewise linear model and for the change in the variance model overlapped (Tables 1–3). It should also be noted that we did not detect any supplementary change point within the elongation zone in the residual series. The residual standard deviation is thus approximately stationary within the elongation zone.

### Consistency of the limit between the elongation zone and the mature zone with the first root hair position

The formation of a root hair bulge is often taken as a marker of the switch from elongation to differentiation. We thus compared for each root the EZ-MZ limit with the first root hair position. For about half of the individuals (16 among 33), the EZ-MZ limit matches with the first root hair position, i.e., the first hair position falls within the uncertainty interval of the EZ-MZ limit or in EZ (Tables 1–3). The situation is contrasting between the wild type and the *rtcs* and *rum-1* mutants since in the case of the mutants, the EZ-MZ limit matches with the first hair position for most of the individuals (7 among 8 for *rtcs* and 7 among 10 for *rum-1*) while this is rather the exception for the wild type (2 among 15 individuals with a least two zones). In particular, the EZ-MZ limit is far from the first hair position in the rootward direction for five wild-type lateral roots of type A.

**Figure 7:**
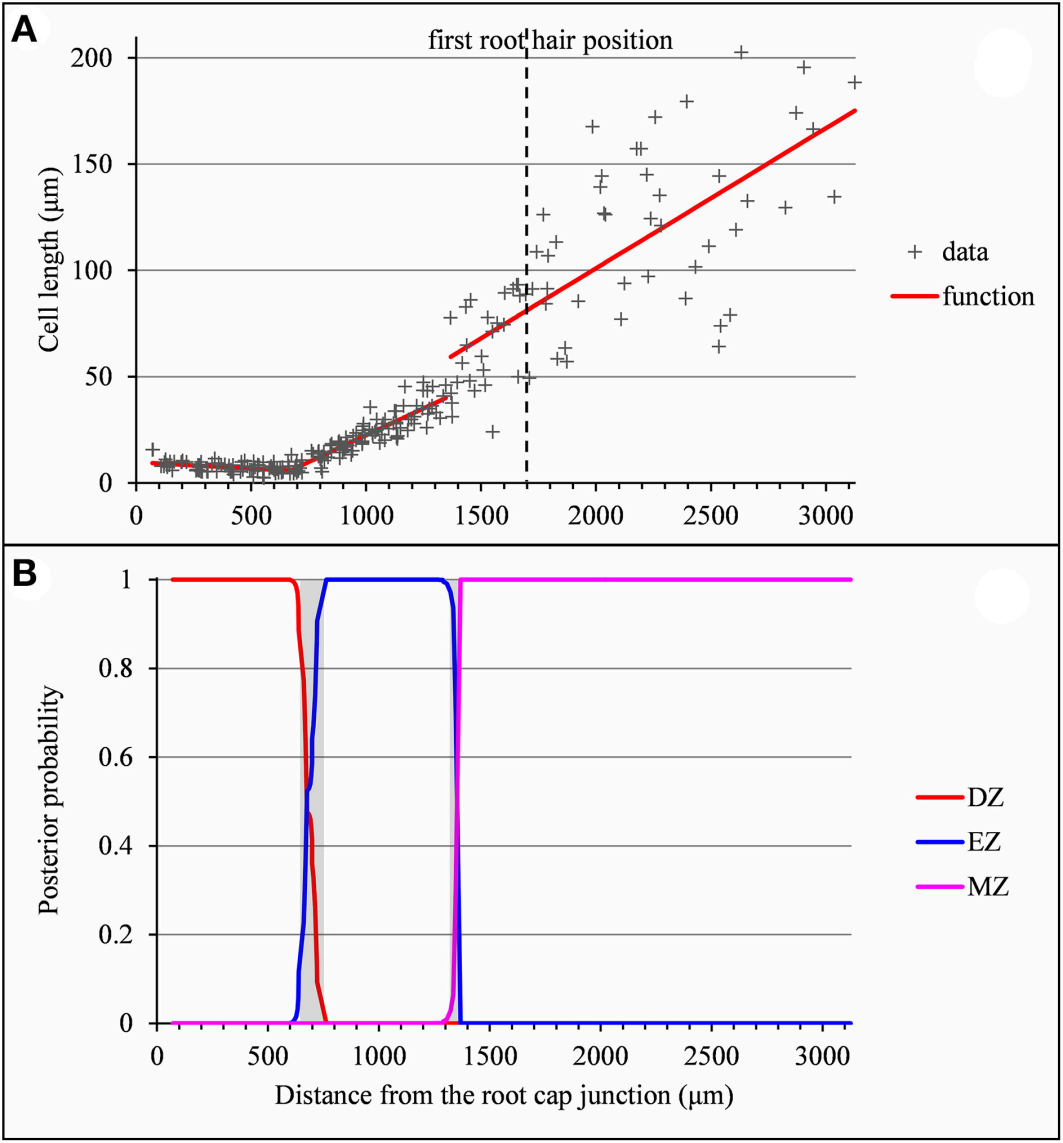
Outputs of the selected piecewise linear model in the case of a lateral root for which the distance between the EZ-MZ limit and the first root hair position is large and the MZ slope is steep (wild-type A31). **(A)** Optimal 3-segment piecewise linear function and first root hair position; **(B)** Posterior division zone (DZ), elongation zone (EZ) and mature zone (MZ) probabilities. The uncertainty intervals for the DZ-EZ and EZ-MZ limits are in gray.

Among the individuals for which the EZ-MZ limit does not match with the first root hair position, we focused on the six 3-zone individuals for which the distance between the EZ-MZ limit and the first root hair position was the largest (wild-type A8, A9, A10, A11, A31, and *rum-1* A6 with a distance between 324 and 1,099 μm); see Table S3. These 6 individuals are also the individuals with the steepest MZ slopes (see Tables 4–6 and Figure 7) and the smallest difference between the MZ slope and the EZ slope among the 3-zone individuals and are characterized by a high overlap between the confidence intervals of the EZ and MZ slopes; see Table S3. It should be noted that for most of the other 3-zone individuals (17 among 20), there is no overlap between the confidence intervals of the EZ and MZ slopes (results not shown). For these 6 individuals, the EZ-MZ limit is thus mainly explained by a change in residual standard deviation. Finally, the cell sampling cannot fully explain these results since for 3 of these individuals, the number of cells beyond the first hair position is above 30 (Table S3). This mismatch of the EZ-MZ limit with the first hair position for some individuals can be viewed as a consequence of the fact that this limit is only explained by a change in residual standard deviation for these individuals while for most individuals, the EZ-MZ limit is explained by a concomitant change in slope and in residual standard deviation. There is a consistent relationship between the EZ-MZ limit and the first root hair position in both the wild type and the two auxin mutants (Figure S4). This relationship is shifted in the mutants with first root hairs emerging closer to the root tip compared to the wild type.

### A strong modulation of the developmental pattern is observed among lateral roots

**Figure 8:**
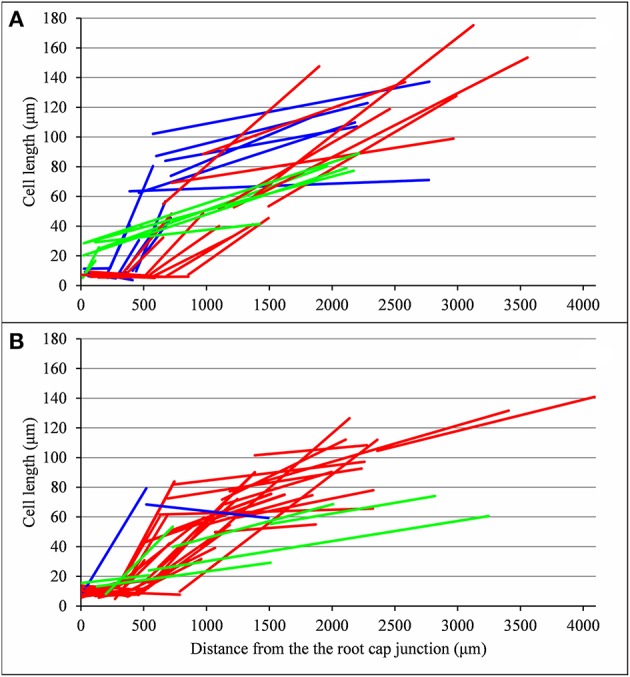
Piecewise linear function estimated for each lateral root (types A, B, and C in red, blue and green respectively): **(A)** wild type, **(B)** *rtcs* and *rum-1* mutants.

As expected from the root sampling strategy, a strong modulation of the developmental pattern is observed among lateral roots (Tables 1–9). While type A roots show the longest division and elongation zones (mean DZ length of 454 μm and mean EZ length of 605 μm), type B roots show much reduced division and elongation zones (mean DZ length of 277 μm and mean EZ length of 284 μm) and type C roots show a lack of division zone and often a lack of elongation zone. Figure 6A illustrates this with a root where a shrunken and no longer active elongation zone is followed by a mature zone with irregular cell length. Figure 6B illustrates a lateral root with neither a division zone, nor an elongation zone but a mature zone with irregularly increasing cell length, possibly the trace of a progressive and irregular deceleration of the root. In both cases, growth arrest is associated with meristem exhaustion and increase in cell length in the apical region. Moreover, DZ cell length is higher in the mutants (6–18 μm; see Tables 8, 9) than in the wild type (4–11 μm, see Table 7). Figure 8 illustrates the diversity of the cell length pattern by showing the piecewise linear functions estimated for each lateral root. It illustrates the ordering of the three types of lateral roots in terms of (i) length of the meristem (from long meristems for type A roots toward absent meristem for most type C roots, type B roots showing shorten meristems), (ii) cell length in MZ (from the longest cells for type A roots to the shortest cells for type C roots). The systematic increase in MZ cell length for type C roots should be noted. This is confirmed by the significant linear correlation coefficients and equivalently the slopes significantly different from zero in the mature zone (Tables 4, 6). Finally, only a limited proportion of potentially growing roots have MZ slopes non-significantly different from zero: nine type A roots (all mutants) among 21 and three type B roots (two wild types and one mutant) among 7 (Tables 4–6). Interestingly, type B roots have the less steep MZ slopes. The variability in MZ slope is also higher for type A roots than for the other types.

### Choice of the variables summarizing lateral root development for the meta-analysis

In order to provide a synthetic view of the modulation of the lateral root developmental pattern, we selected a set of variables for a meta-analysis. The length of the division zone, the length of the elongation zone and the first root hair position were chosen to characterize lateral root development. These three variables are strongly correlated (correlation coefficients between 0.63 and 0.83 for 3-zone lateral roots) which can be interpreted as a longitudinal scaling of lateral root developmental zones. Concerning cell dimension variables, since the elongation zone is the most structuring zone with high estimated correlation coefficients (Tables 4–6), we chose the cell lengths predicted at the two ends of the linear function estimated in the elongation zone for summarizing the change in cell length along the lateral roots. These two predicted cell lengths are positively correlated (*r* = 0.63 for 3-zone lateral roots). There is thus also a scaling effect in the cell length along the roots. The slope within the elongation zone, which is negatively correlated with the length of this zone was also incorporated. Finally, we incorporated the first root hair position and the mean root diameter within the mature zone, in order to explore the relationships between meristem length, growing zone length, root diameter and onset of differentiation.

### Exploration of the diversity of lateral roots using principal components analysis

We applied a principal components analysis (PCA) to the 26 3-zone lateral roots (13 wild-type, 6 *rtcs* and 7 *rum-1* individuals) using 5 variables extracted from the analysis of individual lateral roots using multiple change-point models (DZ length, EZ length, cell length predicted at the DZ-EZ limit, cell length predicted at the EZ-MZ limit, EZ slope) completed by two morphological variables (first root hair position, mean diameter within MZ). We incorporated as supplementary variables in PCA the slope within the division zone. This slope is either negative or non-significantly different from zero for the wild type while being positive (Table 4) or non-significantly different from zero for the *rtcs* mutant (Table 5). The situation of the *rum-1* mutant is intermediate with both positive, negative slopes and slopes non-significantly different from zero (Table 6). We also incorporated as supplementary variables in the PCA the residual standard deviations estimated within each zone. The cell length predicted at the DZ-EZ limit is strongly correlated with the residual standard deviations estimated in DZ and EZ (*r* = 0.8 and *r* = 0.81 respectively) and the cell length predicted at the EZ-MZ limit is strongly correlated with the residual standard deviation estimated in each zone (*r* = 0.58, *r* = 0.77 and *r* = 0.58 for DZ, EZ and MZ respectively).

**Figure 9:**
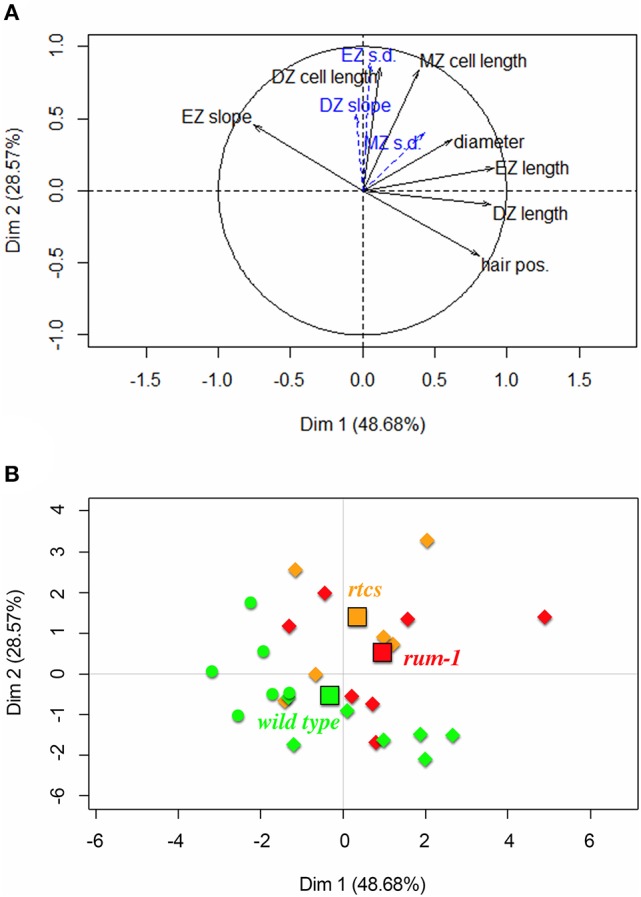
Principal component analysis applied to the 26 3-zone lateral roots: **(A)** Variables factor map with solid black arrows corresponding to variables (division zone, elongation zone and mature zone abbreviated respectively as DZ, EZ, and MZ, first root hair position abbreviated as hair pos., mean diameter within the mature zone abbreviated as diameter; DZ cell length and MZ cell length are shortcuts for cell length predicted at the DZ-EZ limit and at the EZ-MZ limit respectively) used to build the principal components and dotted blue arrows corresponding to supplementary variables (residual standard deviation abbreviated as s.d.); **(B)** Individuals factor map with wild-type individuals in green, *rtcs* individuals in orange and *rum-1* individuals in red (type A roots are indicated by diamonds and type B roots by circles). The genotype centroids are indicated by squares using the same colors.

The first axis accounting for 49% of variance corresponds to the longitudinal variables (mainly DZ and EZ lengths but also first root hair position) while the second axis accounting for 29% of variance corresponds to the cell length variables (cell lengths predicted at the DZ-EZ and the EZ-MZ limits), longitudinal variables and cell length variables being uncorrelated (Figure 9A). All these five variables are highly structuring; see the distances of their projections to the correlation circle. The residual standard deviations within DZ and EZ incorporated as supplementary variables are highly related to the second axis and thus with cell length variables. The EZ slope is less affected by the difference in cell length within EZ (cell length predicted at the EZ-MZ limit – cell length predicted at the DZ-EZ limit) than by the EZ length. Hence, the EZ slope increases when the EZ length decreases; see Figure 9A. Roots from all three genotypes are spread along the first axis but with type B roots being clearly shifted to the left consistently with their smaller DZ and EZ lengths (Figure 9B). Moreover, *rtcs* and *rum-1* individuals are preferentially located above the wild-type individuals, consistently with their DZ and MZ cell length and root diameter being larger.

## Discussion

### Successive developmental zones in the root apex are well characterized by piecewise linear functions

Heretoscedastic piecewise linear models with at most 4 developmental zones were selected for each lateral root with minimum a priori biological assumptions. This result is consistent with the expectation of 3–4 zones for elongating roots: a root apical meristem (Ivanov and Dubrovsky, 2013) possibly including a transition zone, an elongation zone and a mature zone. For non-elongating roots such as type C roots, the lack of both a division zone and an elongation zone is also consistent with the biological knowledge about determinate roots (Dubrovsky and Gómez-Lomeli, 2003; Sánchez-Calderón et al., 2005). These are clear elements of validation of the piecewise linear model assumption but to which extent the linearity assumption matches our biological knowledge within each zone?

Cell length in the apical meristem was well approximated by a single linear function. For only 3 roots among 26, the optimal model induced a split of the apical meristem into 2 zones, the first with a negative slope and the second with a positive or nil slope. Such a split is consistent with the concept of a transition zone where cells progressively leave the cycle while local elongation rate has not yet changed (Dello Ioio et al., 2008; Baluška et al., 2010). Because cell length is the result of an equilibrium between cell division rate and local tissue elongation rate (Green, 1976), a split into 2 zones with negative and positive slopes is consistent with relative elongation rate being constant throughout the meristem (van der Weele et al., 2003) but lower and higher than cell division rate in the two domains respectively (Ivanov et al., 2002) due to more or less cells being engaged in the cycle. The fact that a transition zone was identified in only 3 roots could be due to the short size of this zone (Pacifici et al., 2015) in conjunction with changes in slope and in residual standard deviation of small amplitude.

Beyond these 3 roots, DZ slopes were not systematically zero but rather essentially negative in the wild type while being positive in the *rtcs* mutant and mixed in the *rum-1* mutant. As stated above, a negative slope indicates a proliferative activity being higher than local tissue expansion leading to apparent decrease in cell size (Erickson and Sax, 1956a; Green, 1976; Ivanov et al., 2002). Our results thus suggest that: (i) elongation and division are rarely in perfect equilibrium in the meristem; (ii) in roots from wild-type plants, this equilibrium is in favor of cell division as cells move away from the tip; (iii) deceleration or slow growth is associated with a shortening of the meristem, as classically reported to account for changes in elongation rate of roots exposed to various stresses (Barlow and Adam, 1989; Ryan et al., 1993; West et al., 2004), but not with changes in meristematic cell length; (iv) auxin strongly interferes with the balance between cell division and tissue expansion, at the benefit of the later in the meristem, consistently with the knowledge about the role of this hormone (Pacifici et al., 2015). The outcomes of our model thus essentially fit what is known about the functioning of the root apical meristem and the balance between division and elongation.

Within the elongation zone, the linearity was clear as shown by the high correlation coefficient estimated for most of the lateral roots and the absence of split of this zone. Because the elongation zone corresponds to a zone without cell division, a linear increase in cell length suggests, if elongation of the root is stationary, a linearly increasing local elongation rate and thus a constant relative elongation rate within the elongation zone (Silk et al., 1989). This is what is found in *Arabidopsis* roots when biases due to averaging among individual roots are eliminated (van der Weele et al., 2003). However, the growth of most of our lateral roots was likely non-stationary. Type C roots were already arrested, type B roots were slowly decelerating and type A roots were mostly accelerating with potentially large variability among roots. Our results suggest that even under non-stationary growth regimes, the linear model appears as the most straightforward way to represent the elongation zone in the root apex.

More unexpected was the identification of mature zones often displaying significantly positive slopes as one would not expect from a “classical” mature zone in which cells no longer elongate. A first hypothesis is that this results from non-stationary growth rates, in particular in type A roots. These roots were likely accelerating according to our observations and we know that accelerating roots show increasing mature cell length—e.g., in *Arabidopsis* (Beemster and Baskin, 1998) and in maize (Muller et al., 1998). A possibility is that in these roots, we were observing a transition toward a new growth regime with an elongation zone that progressively increases in length, a first root hair located at an increasing distance from the tip and cells in the mature zone with increasing lengths as classically observed (Beemster and Baskin, 1998; Beemster et al., 2002). Notably, arrested roots also show positive slopes in the mature zone, a result which suggests the opposite situation for decelerating roots, with a progressive decrease of mature cell length as roots decelerate. A second hypothesis is that the mature zone, as identified in our study, is partly a zone with growth deceleration. This hypothesis fits with most classically reported velocity patterns showing progressive deceleration toward the end of the elongation zone in a zone that can occupy a significant length after the zone with fast elongation (see Sharp et al., 1988 in maize, Beemster and Baskin, 1998 in *Arabidopsis*, Bizet et al., 2015 in poplar; Yamaguchi et al., 2010 in soybean). This zone would be longer and therefore its proportion in the mature zone higher for fast growing roots which would explain why MZ slopes in type A roots are globally higher than in type B roots.

Overall, there is a consistent match between the EZ-MZ limit and the first root hair bulge. Because the first root hair bulge is reported to occur rather before the end of elongation, at least in Arabidopsis (Le et al., 2001, 2004; Ma et al., 2003), this further strengthens the second interpretation for the positive slopes detected in MZ using our method. In addition, a distinction could clearly be seen between the wild type and the auxin signaling mutants in agreement with auxin playing also a role—likely in interaction with ethylene; see Ivanchenko et al. (2008) and Cho and Cosgrove (2002)—in coupling cell elongation and differentiation.

### What can be biologically deduced from the identification of developmental zones in roots with non-stationary growth or in arrested roots?

Whereas cell length patterns can be interpreted in a straightforward way in roots with stationary growth (Silk et al., 1989; Fiorani and Beemster, 2006), it becomes more challenging when accelerating, decelerating or arrested roots are considered in particular in the absence of the growth rate profile along the apex. We suggest that the cell length pattern depends on the different time constants for growth rate change, on the one hand, and for cell crossing the apex, on the other hand. Based on Arabidopsis and maize data, we know that cells cross the root meristem in 50–100 h while they cross the elongation zone in a much shorter time (3–10 h; see Muller et al., 1998 for maize data and Beemster and Baskin, 1998; Verbelen et al., 2006 for Arabidopsis data). If we assume that time constant for growth rate changes (i.e., time needed for growth to change by + or −50%) for our maize lateral roots is 1–4 days (type C and B roots typically stop growing within 2–6 days, whereas type A roots never accelerate by more than 50% in 3 days, unpublished data), non-stationary growth would not be an issue when dealing with patterns in the elongation zone or in the mature zone. By contrast, this suggests that a snapshot of a meristem in a root with non-stationary growth gathers cells produced while the root was growing at different rates. The shrinkage of the meristem in type B roots prior to their growth arrest and the lack of meristem in most type C roots fit our knowledge of the functioning of determinate roots (Dubrovsky and Gómez-Lomeli, 2003; Sánchez-Calderón et al., 2005). Type B and C roots would then behave as determinate roots with rapid (type C) or more progressive (type B) meristem shrinkage followed by meristem exhaustion (type C). This pattern of growth cessation in lateral roots thus differs from growth cessation in both monocot and dicot leaves in which, after the end of cell division at the base, all cells elongate reaching the same final length as those of the mature zone (Granier and Tardieu, 1998; Muller et al., 2001; Parent et al., 2009). Despite non-stationary growth, other remarkable results could be observed at the population level. For instance, there was a positive correlation between DZ length and EZ length suggesting that meristem shrinkage occurs in pace with a shortening of the root growing zone.

### How can we interpret the changes in residual standard deviation at the limit between developmental zones?

The limits between successive developmental zones are explained in most cases by a concomitant change in slope and in residual standard deviation. There are at least two reasons for the concomitant change in slope and in residual standard deviation at the DZ-EZ limit. First, epidermal cells differentiate into two distinct types ultimately giving rise to root hair cells (trichoblasts) or non-hair cells (atrichoblasts). While in *Arabidopsis*, these cells are located in distinct cell files (Dolan et al., 1994) and differences in length can be found right at the quiescent center (Beemster and Baskin, 1998), in poaceae as in some other species (Sinnott and Bloch, 1939; Cormack, 1949), trichoblasts and atrichoblasts belong to the same files and differences in length can be seen after the last division at the shootward limit of the meristem which is asymmetrical (Cormack, 1949). Second, the number of rounds of division per cell file in a maize root meristem is very limited, probably no more than 3–4 (Barlow, 1987; Muller et al., 1998) with different cell files being asynchronous (Barlow, 1987). An abrupt increase in dispersion of cell length could thus be due to (i) some cells living the cycle and elongating while others accomplish a last mitosis due to asynchronicity, and (ii) not all cells experiencing exactly the same number of rounds of division. Experiencing 3 or 4 rounds will make a marked difference in terms of length and this difference will show up at the shootward limit of the meristem.

The change in residual standard deviation at the EZ-MZ limit is more challenging to interpret since it occurs after the completion of cell division which is an obvious source of cell length dispersion. Indeed, cells are not supposed to slide from one file to another at a given position from the apex. A possibility is that this abrupt increase is artefactual, due for instance to some cross walls being missed or by contrast attributed to the epidermis whereas they belong to lower layers. However, the concomitant change in slope and in residual standard deviation for a large majority of roots gives little support to this hypothesis. In earlier reports (Goodwin and Stepka, 1945; Pritchard et al., 1990), a massive increase in epidermal cell length dispersion was already reported at the EZ-MZ limit in cereal roots. According to our interpretation, this change also occurs in parallel with growth deceleration. How these developmental events are generated and coordinated deserves further investigations.

### Comparison between segmented regression models and multiple change-points models

Segmented regression or broken-line models are regression models where the regression function is piecewise linear, i.e., made of straight lines connected at change points (Muggeo, 2003). But the homoscedasticity assumption of these models (a residual standard deviation common to the different developmental zones) is very unrealistic in our context. We thus adopted the framework of multiple change-point models which are latent structure models (Guédon, 2013, 2015a) meaning that the outputs of a model are not only the piecewise linear function corresponding to the optimal segmentation but also include the alternative segmentations and more generally various quantities of interest computed on the basis of all the possible segmentations. Contrary to segmented regression models, the piecewise linear functions corresponding to the selected segmentations are not constrained to be continuous in the context of multiple change-point models. This may be viewed as a shortcoming for biological interpretations but the counterparts of choosing the framework of multiple change-points models are numerous: (i) heteroscedastic models can be managed which was mandatory in our context; (ii) the detection of change points is not constrained by the continuity assumption and the approximate continuity is potentially an emerging property of interest; (iii) in multiple-change point models, the inference concerns not only the selection of the number of developmental zones and the estimation of linear function parameters as in standard statistical models such as segmented regression models but also the latent segmentation space (e.g., alternative segmentations). This enables a detailed introspection of each cell length profile with many possibilities to assess biological assumptions.

## Concluding remarks

The proposed method could successfully handle roots with rather strong modulation of the developmental pattern such as arrested roots without division zone or without both division and elongation zones. Our method thus appears both robust and flexible for studying genetic and environmental effects on root development. It is potentially applicable at high throughput, given the possibility to work on epidermal tissues thus avoiding the tedious preparation of longitudinal sections.

Our results highlight a strong coordination of cell division and cell elongation for a large range of fast, slow growing or decelerating lateral roots. As expected, auxin signaling had a marked influence on both coordination between division and elongation in the meristem and between cell growth and differentiation. Our method could thus be used for revisiting the coordination of developmental processes among different cell files within a tissue (e.g., trichoblast, atrichoblast …) or, using longitudinal sections or confocal microscopy, the coordination of developmental processes among different tissues (epidermis, cortex, pericycle, stele …). This could be very useful to extend our knowledge of developmental regulations in longitudinally organized plant organs such as roots, monocot leaves or internodes.

## Author contributions

BMO collected the data, analyzed the data and contributed to the writing of the manuscript, GF collected the data, BM designed the study, analyzed the data and wrote the manuscript, YG designed and implemented the statistical models, analyzed the data and wrote the manuscript.

### Conflict of interest statement

The authors declare that the research was conducted in the absence of any commercial or financial relationships that could be construed as a potential conflict of interest.
