# Supplementary material for: Identifying Developmental Zones in Maize Lateral Root Cell Length Profiles using Multiple Change-Point Models
Source: Front Plant Sci. 2017 Oct 26;8:1750. doi: 10.3389/fpls.2017.01750 (PMC5662930; doi:10.3389/fpls.2017.01750)
Supplement: Supplementary file 1 [file Presentation1.pdf]

Supplementary Material for

Identifying Developmental Zones in Maize Lateral Root Cell Length Profiles using Multiple Change-Point Models

Beatriz Moreno-Ortega[1,2,3,4], Guillaume Fort[1,4], Bertrand Muller[1,4], Yann Guédon[2,3,4]

[1]LEPSE, INRA, Montpellier SupAgro, Montpellier, France

[2] CIRAD, UMR AGAP, Montpellier, France, [3]Inria, Virtual Plants, Montpellier, France

[4]Université de Montpellier, Montpellier, France

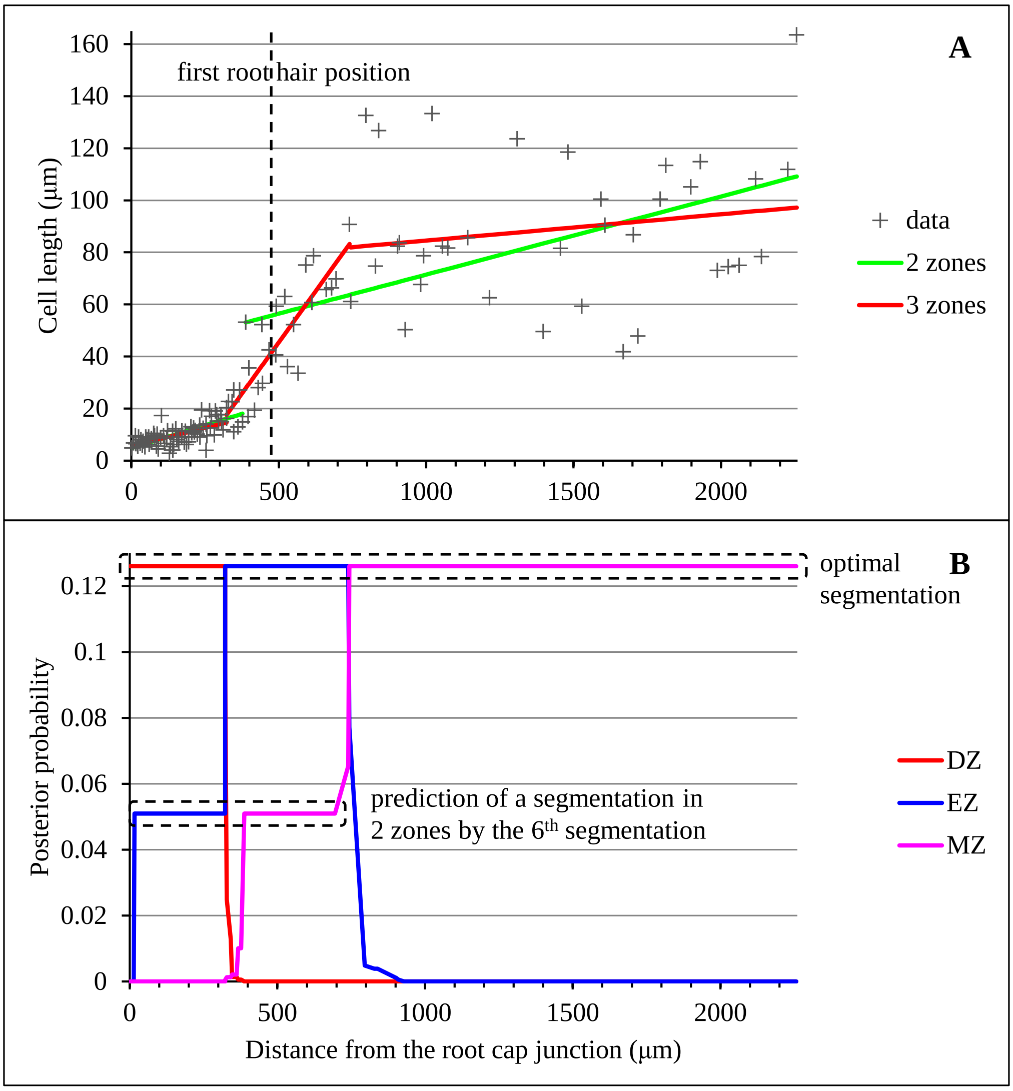

Figure S1. Outputs of the piecewise linear models in the case of a lateral root (*rtcs* A2) for which the 2-zone model selected by the slope heuristic does not fit biological assumptions (lack of the elongation zone). (A) Optimal 2- and 3-segment piecewise linear functions and first root hair position; (B) Posterior segmentation probabilities highlighting the prediction of a 2-zone model by the 6th segmentation in 3 zones −division zone (DZ), elongation zone (EZ) and mature zone (MZ)−.

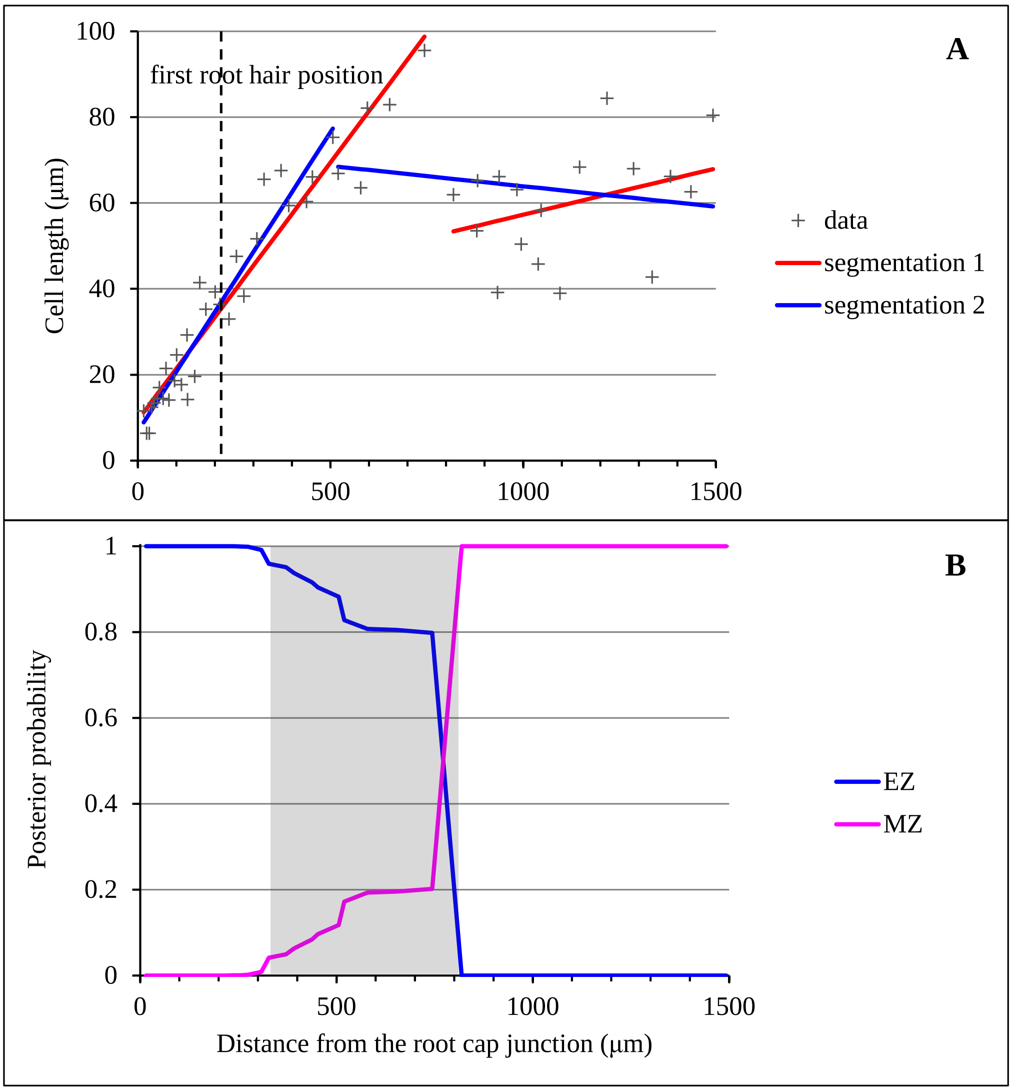

Figure S2. Outputs of the selected piecewise linear model in the case of a probably arrested lateral root (*rtcs* B15) for which the optimal 2-segment piecewise linear function does not fit biological assumptions (piecewise linear function not approximately continuous). (A) Optimal 2-segment piecewise linear function, sub-optimal 2-segment piecewise linear function corresponding to the 2nd segmentation and first root hair position; (B) Posterior elongation zone (EZ) and mature zone (MZ) probabilities. The uncertainty interval for the EZ-MZ limit is in gray.

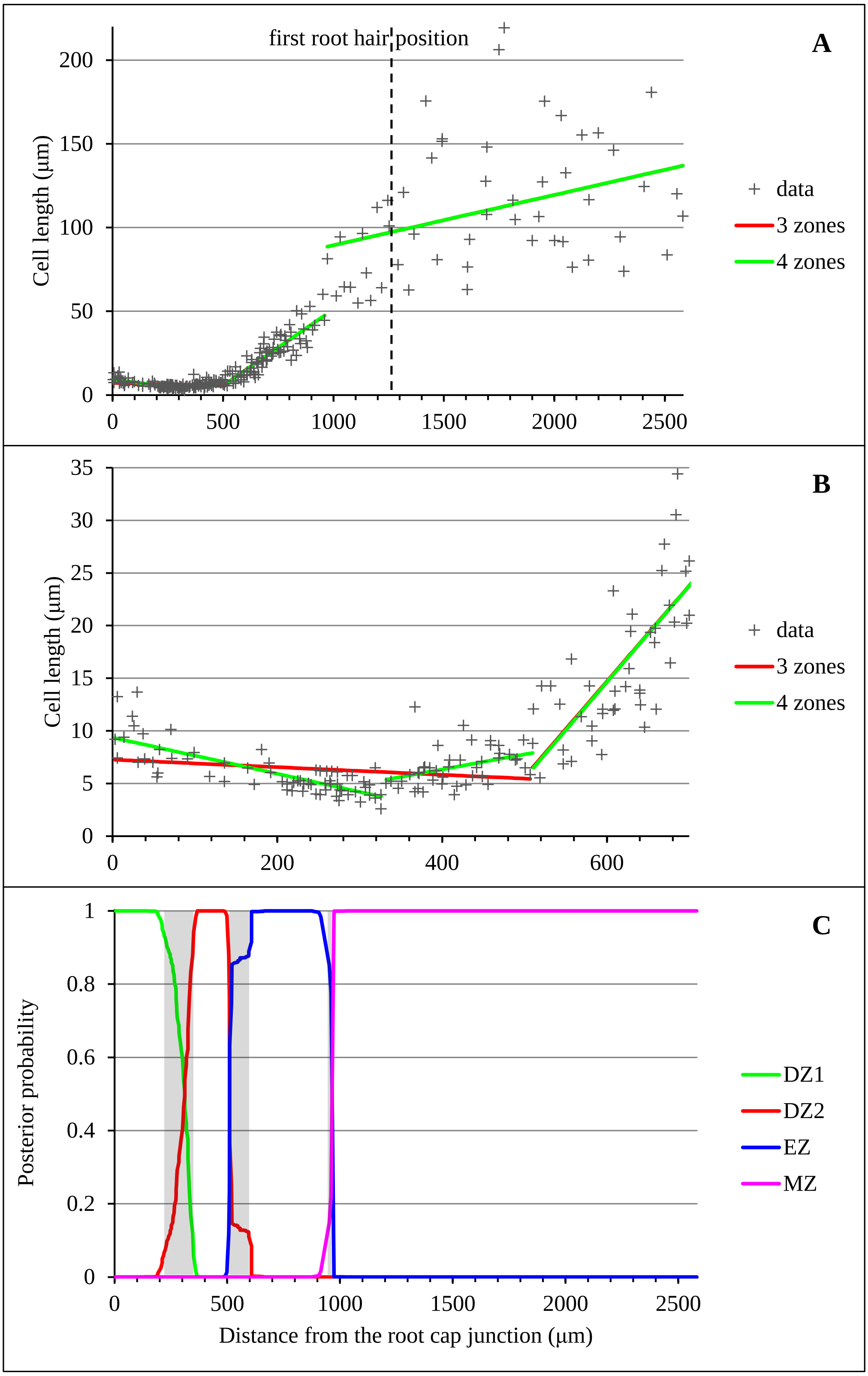

Figure S3. Outputs of the piecewise linear models in the case of a lateral root (wild-type A13) for which 4 zones were identified. (A) Optimal 3- and 4-segment piecewise linear functions and first root hair position; (B) Details of the piecewise linear functions in the division zone; (C) Posterior division zone 1st and 2nd segment (DZ1, DZ2), elongation zone (EZ) and mature zone (MZ) probabilities. The uncertainty intervals for the DZ1-DZ2, DZ2-EZ and EZ-MZ limits are in gray.

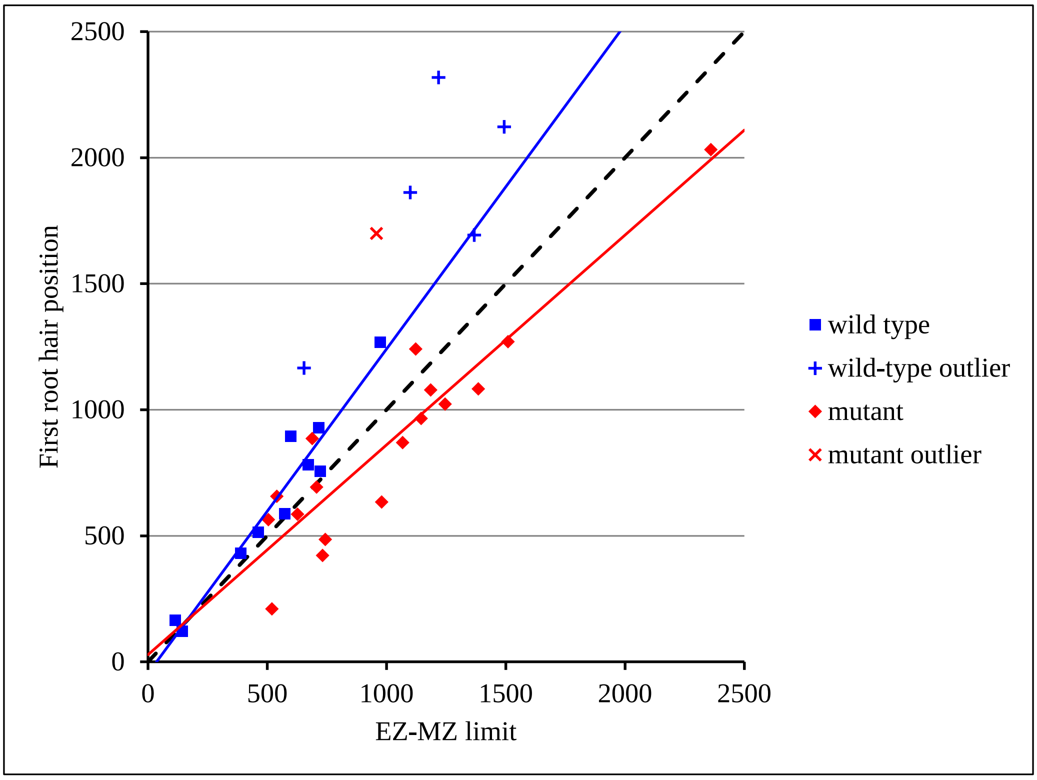

Figure S4. Relationships between the elongation zone-mature zone (EZ-MZ) limit and the first root hair position: The linear trends for wild type and mutants, respectively in blue and red, are computed excluding the 6 outlier individuals (wild-type A8, A9, A10, A11, A31 and *rum-1* A6).

Table S1. Split of the division zone (DZ) for wild-type A13, B33 and B32. The parameters of the first two zones of the selected piecewise linear function (slope x 1000 in µm/mm, correlation coefficient for each zone –n.s. for non-significant−, residual standard deviations –s.d.– and limits between zones –elongation zone (EZ)– in µm with associated 0.05-uncertainty intervals) are given.

	Division zone 1					Division zone 2				
	Slope	Correlation	s.d.	No. cells	DZ1-DZ2 limit	Slope	Correlation	s.d.	No. cells	DZ-EZ limit
A13	−17.2	−0.79	1.4	66	332 (212, 361)	14.3	0.4	1.7	44	511 (501, 608)
B33	−35.4	−0.59	1.2	20	146 (131, 191)	13.3	0.67	1.2	84	439 (428, 451)
B32	−41.4	−0.54	2.6	59	201 (139, 214)	6.2	0.24 n.s.	1	51	411 (340, 411)

Table S2. Split of the division zone (DZ) for wild-type A13, B33 and B32. The first two zones of the selected piecewise linear function (cell lengths predicted at both ends of a zone linked by an arrow and limits between zones –elongation zone (EZ)– in µm) with associated rootward and shootward confidence intervals at each limit between zones (between brackets) are given.

	Division zone 1			Division zone 2		
	Linear function	Confidence intervals	DZ1-DZ2 limit	Linear function	Confidence intervals	DZ-EZ limit
A13	9.3 → 3.7	(3.1, 4.2 | 4.3, 6.4)	332	5.4 → 7.9	(6.8, 9 | 3.8, 9.2)	511
B33	9.3 → 6.2	(4.8, 7.6 | 3.6, 4.6)	146	4.1 → 8	(7.4, 8.6 | 4, 15.1)	439
B32	11.1 → 5	(3.6, 6.5 | 4.6, 5.9)	201	5.3 → 6.6	(5.6, 7.5 | 6.1, 13.8)	411

Table S3. Selection of the six 3-zone individuals the most inconsistent regarding the elongation zone-mature zone (EZ-MZ) limit: difference between the MZ slope and the EZ slope x 1000 (in µm/mm), overlap between the confidence intervals of the EZ and MZ slopes (in % of EZ slope confidence interval), distance between the EZ-MZ limit and the first root hair position (in µm), numbers of cells between the EZ-MZ limit and the first root hair position and beyond the first root hair position.

Genotype	Root	MZ slope − EZ slope	Overlap between EZ and MZ slope confidence intervals	First hair position − EZ-MZ limit	Number of cells: EZ-MZ limit → first hair position	Number of cells: Beyond first hair position
wild type	A8	−9.8	65.3	762	26	11
wild type	A9	0.7	100	1099	32	35
wild type	A10	−9.9	48.4	628	35	33
wild type	A11	−8.4	100	510	34	16
wild type	A31	15.4	45.7	324	25	45
rum-1	A6	30.6	72	742	24	7

Methods S1. Statistical Methods for Heteroscedastic Piecewise Gaussian Linear Models and Gaussian Change in the Variance Models

Let θ denote the set of within-zone parameters (and global mean parameter for Gaussian change in the variance models). For heteroscedastic piecewise Gaussian linear models (M_{linear} models), $\theta = \{\alpha_0, \beta_0, \sigma_0^2, \ldots, \alpha_{J-1}, \beta_{J-1}, \sigma_{J-1}^2\}$ while for Gaussian change in the variance models (M_{variance} models), $\theta = \{\alpha, \sigma_0^2, \ldots, \sigma_{J-1}^2\}$. Let $f_J(\mathbf{s}, \mathbf{x}; \hat{\theta})$ denote the likelihood of the segmentation **s** in *J* developmental zones of the observed cell length series $\mathbf{x} = x_1, \ldots, x_T$. The estimation of the $J-1$ change points $\tau_1, \ldots, \tau_{J-1}$, which corresponds to the optimal segmentation $\mathbf{s}^*$ into *J* developmental zones, is obtained as follows

$$\hat{\tau}_1, \ldots, \hat{\tau}_{J-1} = \arg\max_{\mathbf{s}} \log f_J(\mathbf{s}, \mathbf{x}; \hat{\theta}),$$

with

$$\log f_J(\mathbf{s}, \mathbf{x}; \hat{\theta}) = -\sum_{j=0}^{J-1} \frac{\tau_{j+1} - \tau_j}{2} \left[\log \left\{ \frac{\sum_{t=\tau_j}^{\tau_{j+1}-1} (x_t - \hat{\alpha}_j - \hat{\beta}_j t)^2}{\tau_{j+1} - \tau_j} \right\} + \log(2\pi) + 1 \right] \quad \text{for } M_{\text{linear}} \text{ model,}$$

$$\log f_J(\mathbf{s}, \mathbf{x}; \hat{\theta}) = -\sum_{j=0}^{J-1} \frac{\tau_{j+1} - \tau_j}{2} \left[\log \left\{ \frac{\sum_{t=\tau_j}^{\tau_{j+1}-1} (x_t - \hat{\alpha})^2}{\tau_{j+1} - \tau_j} \right\} + \log(2\pi) + 1 \right] \quad \text{for } M_{\text{variance}} \text{ model.}$$

For this optimization task, the additivity in *j* of the maximized log-likelihoods for each zone, allows us to use a dynamic programming algorithm (Auger and Lawrence, 1989) whose computational complexity is $O(JT^2)$ in time.

Regarding the inference of multiple change-point models, one key question is to select the number of developmental zones. In a model selection context, the purpose is to estimate *J* by maximizing a penalized version of the log-likelihood defined as follows

$$\hat{J} = \arg\max_{J} \{\log f_J(\mathbf{x}) - \text{Penalty}(J)\},$$

where

$$f_J(\mathbf{x}) = \sum_{\mathbf{s}} f_J(\mathbf{s}, \mathbf{x}; \hat{\theta})$$

is the log-likelihood of all the possible segmentations in *J* developmental zones of the observed cell length series **x** of length *T*. The principle of this kind of penalized likelihood

criterion consists in making a trade-off between an adequate fitting of the model to the data (expressed by the log-likelihood) and a reasonable number of parameters to be estimated (controlled by the penalty term). The most popular information criteria such as AIC and BIC are not adapted in this particular context since they tend to underpenalize the log-likelihood and thus select a too large number of developmental zones (Rigaill et al., 2016). We thus applied the slope heuristic (SH) given by (Guédon, 2015)

$$\mathrm{SH}_J = 2\left\{\log f_J(\mathbf{x}) - 2\hat{\kappa}\,\mathrm{pen}_{\mathrm{shape}}(J)\right\},$$

where

$$\mathrm{pen}_{\mathrm{shape}}(J) = \log\left\{\frac{T^{J-1}}{(J-1)!}\right\},$$

and $\hat{\kappa}$ is the slope of the linear relationship between $\log f_J(\mathbf{x})$ and $\mathrm{pen}_{\mathrm{shape}}(J)$ for overparameterized models estimated by the data-driven slope estimation method (Baudry et al., 2012). The posterior probability of the *J*-developmental-zone model M_J, given by

$$P(M_J \mid \mathbf{x}) = \frac{\exp\left(\frac{1}{2}\mathrm{SH}_J\right)}{\sum_{K=1}^{J_{\max}} \exp\left(\frac{1}{2}\mathrm{SH}_K\right)},$$

can be used to assess the relative merits of the models considered.

The posterior probability of the optimal segmentation $\mathbf{s}^*$ given by

$$P(\mathbf{s}^* \mid \mathbf{x}; J) = f_J(\mathbf{s}^*, \mathbf{x}; \hat{\theta}) / \sum_{\mathbf{s}} f_J(\mathbf{s}, \mathbf{x}; \hat{\theta}),$$

can be efficiently computed by the smoothing algorithm proposed by Guédon (2013). The assessment of multiple change-point models thus relies on two posterior probabilities:

- posterior probability of the *J*-developmental-zone model M_J, $P(M_J \mid \mathbf{x})$ deduced from the slope heuristic computed for a collection of multiple change-point models for $J = 1, \ldots, J_{\max}$, i.e. weight of the *J*-developmental-zone model among all the possible models between 1 and $J_{\max}$ developmental zones,
- posterior probability of the optimal segmentation $\mathbf{s}^*$ for a fixed number of developmental zones J $P(\mathbf{s}^* \mid \mathbf{x}; J)$, i.e. weight of the optimal segmentation among all the possible segmentations for a fixed number of developmental zones.

It is often of interest to quantify the uncertainty concerning change-point position. To this end, we computed the posterior change-point probabilities for each change point *j* and each position *t* using the smoothing algorithm proposed by Guédon (2013). We define the α-uncertainty interval for change point *j* as the interval such that

$$\alpha/2 < \sum_{t=u}^{v} P(S_t = j, S_{t-1} = j-1 \mid \mathbf{x}; J) < 1-\alpha/2,$$

with $\sum_{t=j+1}^{\tau-J+j} P(S_t = j, S_{t-1} = j-1 \mid \mathbf{x}; J) = 1$. In this uncertainty interval, $P(S_t = j-1 \mid \mathbf{x}; J)$ is monotonically decreasing as a function of *t* while $P(S_t = j \mid \mathbf{x}; J)$ is monotonically increasing and $P(S_t = j \mid \mathbf{x}; J) = 1 - P(S_t = j-1 \mid \mathbf{x}; J)$ if there is no overlap between uncertainty intervals for consecutive change points; see illustrations in Figs 2b, 3b, 4b, 5b, 7b, S2b and S3c.

Other posterior probability profiles of interest can be obtained using the forward-backward dynamic programming algorithm (Guédon, 2013). Rather than summarizing all the possible segmentations as in the posterior zone probability profiles $\left\{P(S_t = j \mid \mathbf{x}; J); j = 0,\ldots,J-1; t = 1\ldots,T\right\}$, the idea is to highlight structural differences between alternative segmentations and the optimal segmentation by computing

$$\left\{\max_{s_1,\ldots,s_{t-1}} \max_{s_{t+1},\ldots,s_T} P(S_1 = s_1,\ldots,S_{t-1} = s_{t-1}, S_t = j, S_{t+1} = s_{t+1},\ldots,S_T = s_T \mid \mathbf{x}; J); j = 0,\ldots,J-1; t = 1\ldots,T\right\}$$

These posterior segmentation probability profiles are illustrated in Figs 4c, 5c and S1b.

References

Auger I. E., and Lawrence, C. E. (1989). Algorithms for the optimal identification of segment neighborhoods. *Bulletin of Mathematical Biology* 51, 39-54.

Baudry, J.-P., Maugis, C., and Michel, B. (2012). Slope heuristics: overview and implementation. *Statistics and Computing* 22(2), 455-470.

Guédon, Y. (2013). Exploring the latent segmentation space for the assessment of multiple change-point models. *Computational Statistics* 28(6), 2641-2678.

Guédon, Y. (2015). Slope heuristics for multiple change-point models. In: *30th International Workshop on Statistical Modelling* (IWSM 2015), Linz, Austria. Friedl H, Wagner H. eds., vol. 2, 103-106.

Rigaill, G., Lebarbier, E., Robin, S. (2012). Exact posterior distributions and model selection criteria for multiple change-point detection problems. *Statistics and Computing* 22(4), 917-929.
